# Emerging applications of gene editing technologies for the development of climate-resilient crops

**DOI:** 10.3389/fgeed.2025.1524767

**Published:** 2025-03-10

**Authors:** R. L. Chavhan, S. G. Jaybhaye, V. R. Hinge, A. S. Deshmukh, U. S. Shaikh, P. K. Jadhav, U. S. Kadam, J. C. Hong

**Affiliations:** ^1^ Vilasrao Deshmukh College of Agricultural Biotechnology, Vasantrao Naik Marathwada Krishi Vidyapeeth, Latur, India; ^2^ Division of Applied Life Science (BK21 Four), Division of Life Science, Plant Molecular Biology and Biotechnology Research Centre (PMBBRC), Gyeongsang National University, Jinju, Republic of Korea

**Keywords:** genome editing, climate-resilience crops, abiotic and biotic stress, climate change, CRISPR-cas (clustered regularly interspaced short palindromic Repeats-CRISPRassociated) system, base editing (BE)

## Abstract

Climate change threatens global crop yield and food security due to rising temperatures, erratic rainfall, and increased abiotic stresses like drought, heat, and salinity. Gene editing technologies, including CRISPR/Cas9, base editors, and prime editors, offer precise tools for enhancing crop resilience. This review explores the mechanisms of these technologies and their applications in developing climate-resilient crops to address future challenges. While CRISPR/enables targeted modifications of plant DNA, the base editors allow for direct base conversion without inducing double-stranded breaks, and the prime editors enable precise insertions, deletions, and substitutions. By understanding and manipulating key regulator genes involved in stress responses, such as *DREB, HSP, SOS, ERECTA, HsfA1,* and *NHX;* crop tolerance can be enhanced against drought, heat, and salt stress. Gene editing can improve traits related to root development, water use efficiency, stress response pathways, heat shock response, photosynthesis, membrane stability, ion homeostasis, osmotic adjustment, and oxidative stress response. Advancements in gene editing technologies, integration with genomics, phenomics, artificial intelligence (AI)/machine learning (ML) hold great promise. However, challenges such as off-target effects, delivery methods, and regulatory barriers must be addressed. This review highlights the potential of gene editing to develop climate-resilient crops, contributing to food security and sustainable agriculture.

## Introduction

The increase in global population and severe climate change are primary challenges to food security. Higher greenhouse gas emissions lead to increased atmospheric temperatures. It is projected that an average increase of 2°C by the year 2100 (Scafetta, 2024). This will cause substantial economic losses in agriculture and food production (Abdelrahman et al., 2021). Climate change presents abiotic stresses via salinity, drought, and temperature stress that affect crop physiology, reduce productivity, and threaten global food security (Abdelrahman et al., 2021) Moreover, climate change negatively impacts soil microbial populations and their enzymatic functions, particularly in arid regions (Cooper et al., 2018). It alters plants’ physiological and metabolic processes, affecting growth, pest dynamics, and agricultural productivity. These factors raise the risk of pest invasions and plant diseases, exacerbating fragile food production (Abdelrahman et al., 2021).

Rapid and unpredictable changes in climatic conditions command novel solutions to develop resilient crops that can withstand the challenges of extreme drought, heat, and salinity. Gene editing technologies like CRISPR/Cas9, transcription activator-like effector nucleases (TALENs), zinc finger nucleases (ZFNs), and base editors provide a significant opportunity for crop improvement (Biswas et al., 2021). These methods allow for precise, targeted modifications in a plant’s genetic makeup, facilitating the development of crops with enhanced traits, including increased yield, pest and disease resistance, and improved resilience to environmental stresses (Ahmad A. et al., 2021) Gene editing holds immense potential for addressig global agricultural challenges, including improved climate resilience, enhanced nutritional content, increased yield and productivity, pest and disease resistance, and faster breeding cycles (Biswas et al., 2021; Kumar et al., 2017).

## Gene editing technologies

### CRISPR/Cas9

CRISPR/Cas9 was initially discovered in *Escherichia coli* (Ishino et al., 1987). CRISPR/Cas functions as an adaptive immune defense in bacteria, safeguarding their genomic DNA from viral (phage) attacks by degrading the DNA with the help of RNA guidance and the Cas9 protein (Mallapaty, 2019; Li et al., 2020a). CRISPR/Cas systems are classified into two main classes (Jinek et al., 2012). Class 1 systems require multiple effector proteins, whereas Class 2 systems use a single protein. Class 1 is further divided into types I, III, and IV, while Class 2 includes types II, V, and VI (Makarova et al., 2011; 2015). Type II systemsprimarily use Cas9 and are the most widely applied (Wiedenheft et al., 2012; Sorek et al., 2013). The CRISPR/Cas9 mechanism incorporates fragments of foreign DNA into the CRISPR locus, which are later transcribed into CRISPR RNA (crRNA). This crRNA binds with trans-activating crRNA (tracrRNA), enabling Cas9 to identify and cleave the target foreign DNA sequence (Jinek et al., 2012).

Cas9 requires the presence of a conserved protospacer-adjacent motif (PAM) sequence upstream of the crRNA binding region to accurately identify the target sequence (Jinek et al., 2012). The CRISPR/Cas9 complex is composed of the Cas9 endonuclease, trans-activating crRNA (tracrRNA), CRISPR RNA (crRNA), and RNase III (El-Mounadi et al., 2020; Mohanta et al., 2017; Jansing et al., 2019). The tracrRNA and crRNA can be combined into a single-guide RNA (sgRNA), which incorporates elements of both (Kumar et al., 2019). Cas9, which cleaves double-stranded DNA (dsDNA), has two active domains: The His-Asn-His (HNH) and RuvC-like domains. These domains cut the dsDNA three base pairs upstream of the PAM sequence (5′-NGG or 5′-NAG) (Jiang and Doudna, 2017; Hille and Charpentier, 2016; Manghwar et al., 2019). The HNH domain cleaves the strand complementary to the crRNA, while the RuvC-like domain cleaves the opposite strand, creating double-stranded breaks (DSBs) that are repaired via non-homologous end joining (NHEJ) or homology-directed repair (HDR) (Kumar et al., 2019; Jiang and Doudna, 2017).

The sgRNA is approximately 100 nucleotides long and includes a 20-nucleotide guide sequence at the 5′end, which directs the complex to the target site, followed by the PAM sequence. The 3′end of the sgRNA forms a loop structure that aids in binding to the target DNA. Together, the sgRNA and Cas9 create a ribonucleoprotein (RNP) complex capable of cleaving the target DNA. The crRNA plays a key role in recognizing the target DNA and assists the RNP complex in binding by forming an R-loop-like structure (Manghwar et al., 2019). The loop structure formation activates both active domains of the Cas9 endonuclease, resulting in the cleavage of double-stranded DNA (dsDNA) and the production of blunt ends (Hille and Charpentier, 2016). For *Streptococcus pyogenes* Cas9, the recognized PAM sequence is typically 5′-NGG-3′, although 5′-NAG-3′ is sometimes tolerated (Hsu et al., 2013; Jiang et al., 2013; Mali et al., 2013).

Research has shown that the guide RNA (gRNA) forms a heteroduplex with the complementary DNA strand within a positively charged groove situated between the HNH and RuvC-like domains of Cas9 (Lu et al., 2022). An arginine-rich motif in Cas9 is essential for PAM recognition (Anders et al., 2014). It is found that the displacement of the DNA strand triggers a conformational shift in Cas9, positioning the non-target DNA strand within the RuvC domain and moving the HNH domain closer to the target strand, thus facilitating the cleavage of both strands (Jiang et al., 2016). This adaptability allows CRISPR/Cas systems to create unidirectional double-stranded breaks in DNA efficiently. Such breaks activate cellular DNA repair pathways, introducing precise mutations at the target site.

**FIGURE 1:**
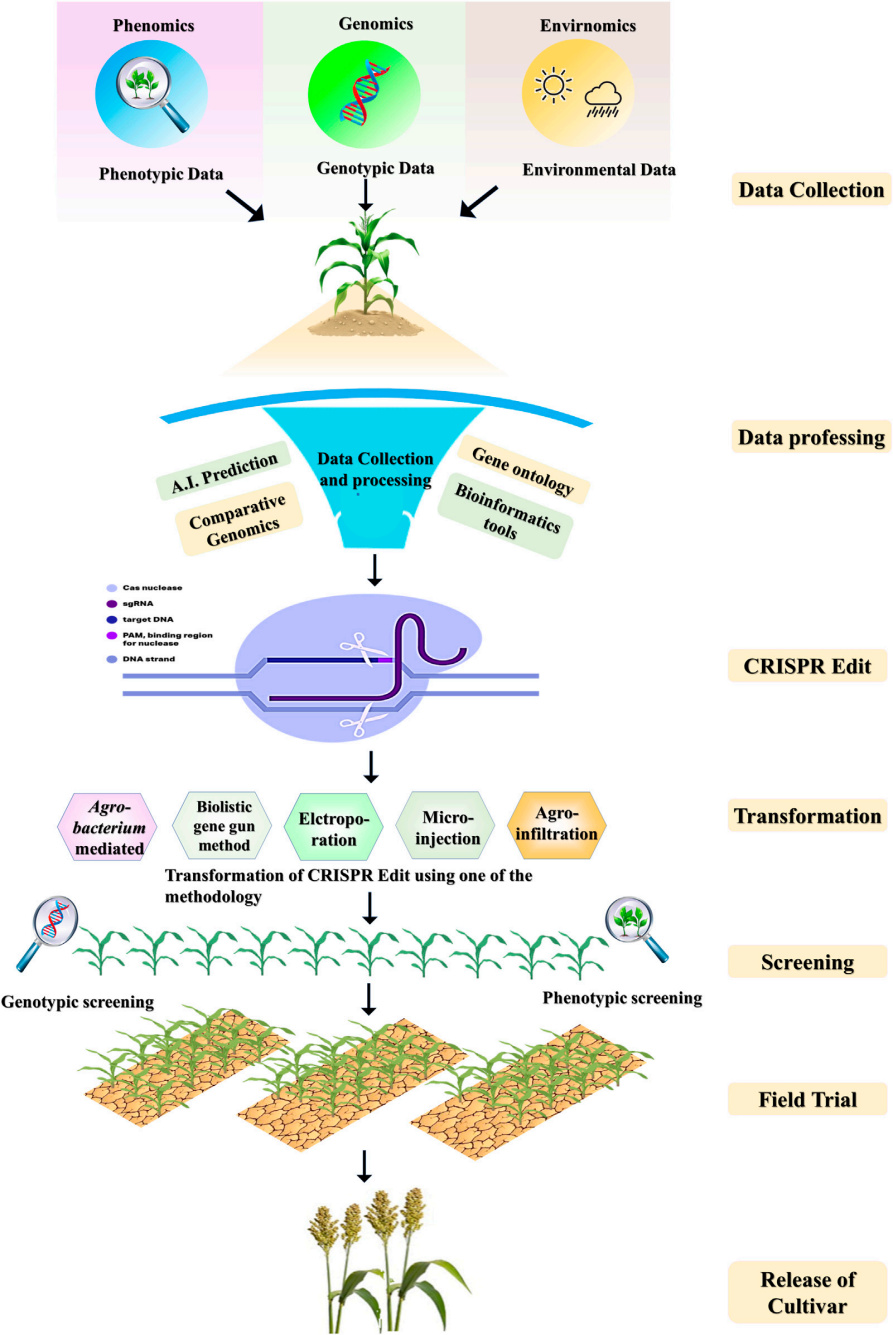
Diagrammatic representation of CRISPR-Cas and its workflow.

This method is widely used for gene knockout by inducing insertions and deletions (INDELs) through the nonhomologous end joining (NHEJ) repair pathway. Alternatively, when a donor template similar to the target DNA is available, genes can be integrated or corrected via the homology-directed repair (HDR) pathway (Gaj et al., 2016). Since the gRNA facilitates target recognition, CRISPR/Cas9 serves as a powerful genome editing tool, eliminating the need to design custom proteins for each target site. Its ease of programming, precise cutting capability, and versatile system variants have driven significant progress in the field (Figure 1). This cost-effective and user-friendly technology enables precise targeting, editing, modification, regulation, and labeling of genomic loci across various cell types and organisms (Doudna and Charpentier, 2014). CRISPR/Cas9 has yielded remarkable achievements, including improvements in nutritional content (Li A. et al., 2018), the creation of male-sterile maize (Li et al., 2017) and wheat (Okada et al., 2019), the development of disease-resistant crops (Zhang et al., 2017), and the production of herbicide-resistant plants (Sun et al., 2016). Notably, in 2021, Japan introduced the world’s first CRISPR/Cas9-edited tomato*, i.e., Sicilian Rouge High GABA tomato* (Waltz, 2022)*,* engineered to have increased levels of γ-aminobutyric acid (GABA) (Ezura, 2022).

**FIGURE 2:**
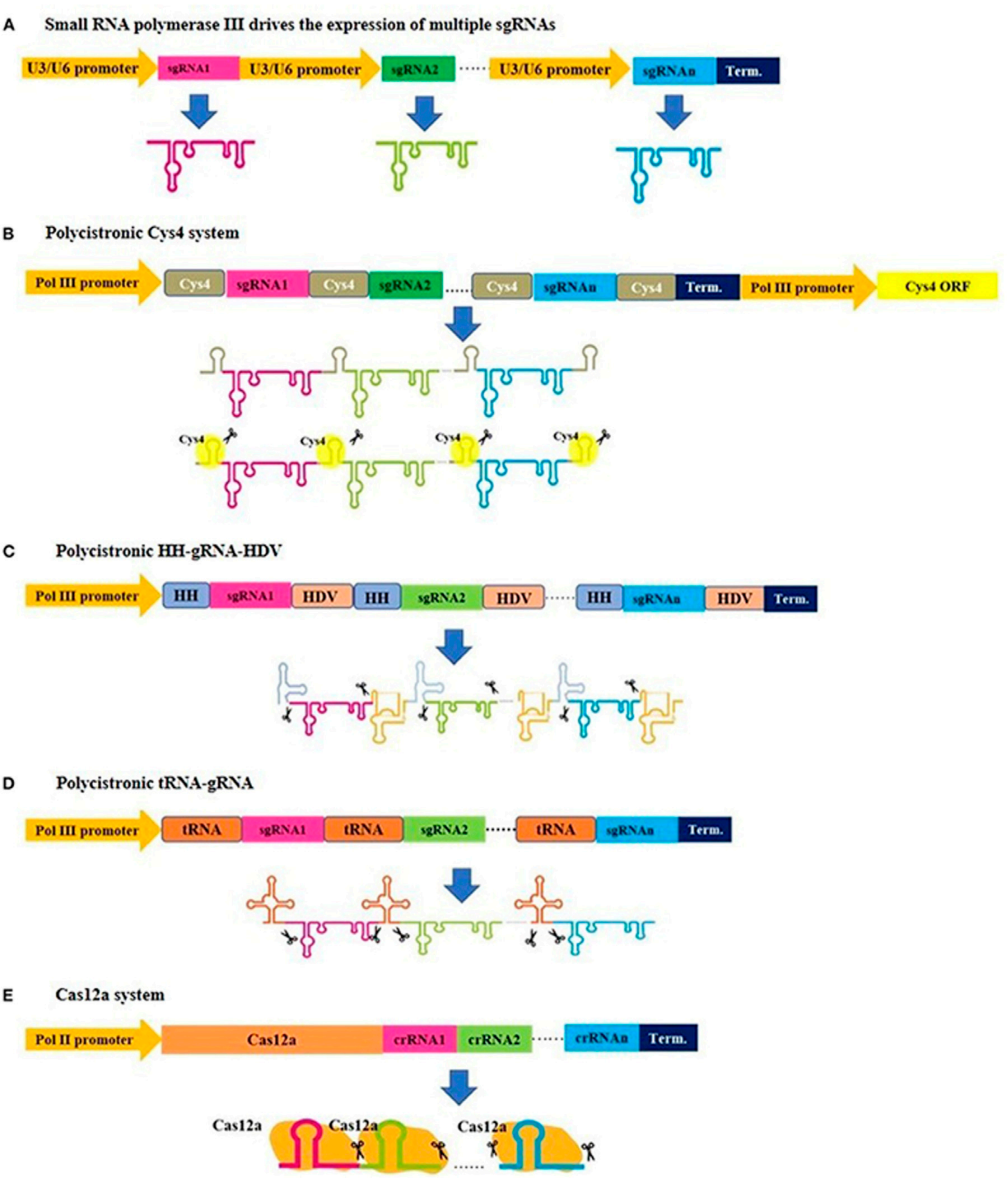
An illustration demonstrating various strategies for expressing multiplex gRNA cassettes in plants. **(A)** Small gRNAs are cloned after U3 or U6 promoters and derived by small RNA polymerase III to generate individual gRNAs. **(B–D)** Small gRNAs are cloned to be transcribed into a single transcript, and subsequent posttranscriptional processing is needed for gRNA separation, where Csy4, tRNAs, and hammerhead ribozyme regulate this separation. Similarly, a single transcript is generated in the **(E)** Cas12a system, but this system has a gRNA self-cleaving feature and does not require additional elements for posttranscriptional processing. (Figure from Abdelrahman et al., 2021; Copyright: CC BY License).

CRISPR/Cas9 technology holds a significant edge over zinc finger nucleases (ZFNs) and transcription activator-like effector nucleases (TALENs). The CRISPR/Cas9 system employs a programmable single-guide RNA (sgRNA) for sequence-specific DNA targeting, enabling unprecedented precision in genome editing through its complementarity-driven recognition mechanism, which significantly reduces molecular complexity compared to traditional gene-editing approaches (Doggalli et al., 2024). This has accelerated advancements in plant breeding and molecular research. A key advantage of CRISPR/Cas9 is its multiplexing capability, allowing simultaneous targeting of multiple genes and facilitating the rapid development of complex trait combinations (Figure 2). This feature is especially valuable for engineering disease resistance and studying gene interactions (Goberna et al., 2022). Additionally, its high efficiency and potential for creating transgene-free crop varieties have made it an indispensable tool for developing plants with enhanced traits, such as higher yields, improved quality, and increased disease resistance (Zhang and Qi, 2019). CRISPR/Cas9 also has limitations, notably the risk of off-target effects, where the Cas9 enzyme may cleave similar but unintended DNA sequences. This can result in unexpected gene structure and function changes, potentially leading to undesirable phenotypes. To mitigate these risks, researchers have developed strategies such as improving sgRNA design, using truncated or modified sgRNAs, exploring Cas9 mutants and orthologues, and employing the “double nicking” strategy. Applying CRISPR/Cas9 technology to crop improvement faces several challenges, including delivery issues and regulatory hurdles. Addressing these challenges is crucial for advancing CRISPR/Cas9 technology in plant biotechnology and fully realizing its potential in crop improvement.

### Base editors

Base editing technology is an advanced genome editing method derived from the CRISPR/Cas9 system. It empowers precise single-base substitutions without inducing double-strand breaks or requiring donor DNA templates (Molla et al., 2021; Negishi et al., 2019). This method employs engineered deaminases to convert specific nucleotides, such as cytosine to thymine (C-to-T) or adenine to guanine (A-to-G), enabling targeted genetic modifications with high efficiency and specificity (Huang et al., 2023). Unlike traditional CRISPR/Cas9, which often results in insertions or deletions (indels) due to DSB repair, base editing yields more predictable outcomes, making it particularly beneficial for plant genome engineering (Li et al., 2023). The mechanism involves a modified CRISPR/Cas9 system, where a catalytically inactive Cas9 protein is fused to an active deaminase enzyme. The process begins with a single-guide RNA (sgRNA) directing the Cas9 protein to a specific genomic location (Yang X. et al., 2024). Once bound, the Cas9 creates a single-strand break, allowing the deaminase to access the target base. Depending on the base editor used—either a cytidine base editor (CBE) or an adenine base editor (ABE)—the deaminase converts cytidine (C) to uracil (U) or adenine (A) to inosine (I). During DNA replication, these modifications result in the desired C ⋅ G to T ⋅ A or A ⋅ T to G ⋅ C substitutions (Azameti and Dauda, 2021).

Base editing systems consist of molecular components that function together effectively. Cytosine base editors (CBEs) typically include a deaminase (variants of DddA or APOBEC) fused to TALE arrays for targeted binding, enabling C-to-T conversions (Wang X. et al., 2024; Zhang D. et al., 2024). Recent findings like the TadA-8e-derived CBEs and mTCBE variants have achieved editing efficiencies of up to 81% in rice (Wang Y.-H. et al., 2024; Fan, 2024). The A3A-CBE variant offers a broad editing range, effectively targeting multiple sites simultaneously (Luo et al., 2023). Adenine base editors (ABEs) are dCas9 linked to an adenine deaminase, facilitating A-to-G conversions. For example, the ABE8e variant has demonstrated enhanced editing capabilities in various crops (Fan, 2024; Arantes et al., 2024). Additionally, dual base editors have been developed, combining both CBE and ABE functionalities to enable simultaneous editing of cytosines and adenines, thereby expanding the potential for genetic modifications in plants (Fan et al., 2024; Wang X. et al., 2024).

The alteration of GmAITR genes, leading to*gmaitr36* double and *gmaitr23456* quintuple mutants in soybean using CRISPR/Cas9, has shown enhanced salinity tolerance, highlighting base editing’s potential to improve abiotic stress responses (Wang J. et al., 2021). Moreover, base editing technologies have demonstrated high efficiency and specificity in various crops, including rice and Arabidopsis, allowing for the precise development of desirable traits compared to conventional breeding techniques (Ren et al., 2021; Lyzenga et al., 2021). Recent studies have successfully introduced herbicide resistance and created beneficial mutations in crops, showcasing the practical applications of these systems in agricultural biotechnology (Fan et al., 2024; Zhong et al., 2024). Such precision accelerates the breeding process and helps address challenges related to genetic diversity and linkage drag common in traditional methods. Additionally, the capability for simultaneous multiple edits enhances its utility in crop improvement, enabling the development of varieties with desirable traits such as disease resistance and improved nutritional content (Azameti and Dauda, 2021). Despite its advantages, challenges persist in optimizing editing ranges and enhancing the efficiency of specific base conversions, especially in diverse plant species (Zhong et al., 2024). Additionally, the potential for off-target effects requires further optimizing these systems (Zhang W. et al., 2024). Overall, base editing marks a significant advancement in the toolkit for crop improvement and functional genomic research (Sun et al., 2024; Li et al., 2023).

Adenine base editors (ABEs) are engineered tools comprising nCas9 (D10A) fused with an artificially evolved adenosine deaminase, which catalyzes the conversion of adenine (A) to inosine (I), subsequently leading to A:T to G:C base substitutions during DNA repair and replication (Gaudelli et al., 2017). The initial ABE7.10 construct was developed by fusing nCas9 (D10A) with a heterodimer of wild-type adenine deaminase TadA and an evolved variant TadA7.10, enabling an editing window spanning positions 4–8 nt in the protospacer region, with the PAM located at positions 21–23 nt. Enhancements in editing efficiency were achieved by optimizing the codon usage and incorporating an additional nuclear localization sequence (NLS) for use in mammalian cells (Koblan et al., 2018). To further improve performance, ABEmax was developed by adding NLS motifs at both termini of ABE7.10, resulting in editing efficiencies below 50% at most target loci (Hua et al., 2018; Li C. et al., 2018; Yan et al., 2018). ABEmax facilitated A: T to G:C conversions at loci such as *OsACC*, *OsMPK6*, *OsSERK2*, and *OsWRKY45* in rice with editing frequencies of 17.6%, 32.1%, and 62.3%, respectively (Yan et al., 2018). Additionally, a simplified ABE variant, ABE-P1S (TadA7.10-nCas9 D10A), demonstrated superior editing efficiency in rice compared to the commonly used TadATadA7.10-nCas9 D10A fusion (Hua et al., 2020).

Subsequently, ABE8e was created by incorporating TadA8e, a more efficient adenine deaminase variant that evolved from TadA7.10 (Gaudelli et al., 2020; Richter et al., 2020). ABE8e exhibited a significantly higher deamination rate, enhancing A-to-G conversion efficiency (Richter et al., 2020). A V106W mutation in TadA8e was introduced to minimize off-target effects (Richter et al., 2020). A rice-optimized version, rABE8e, was later developed by combining codon-optimized monomeric TadA8e with bis-bpNLS (dual NLSs at both termini), leading to markedly improved editing efficiencies on NG-PAM and NGG-PAM target sequences compared to ABEmax in rice. rABE8e achieved near-complete editing efficiencies and a higher homozygous substitution ratio within the editing window, particularly at positions A5 and A6 (Wei et al., 2021).

Recently, the ABE toolbox was further advanced with the development of PhieABE, which integrates hyTadA8e (TadA8e with a single-stranded DNA-binding domain) to achieve significantly enhanced base editing activity and expanded editing windows compared to standard ABE8e systems (Tan et al., 2022). Finally, TadA9, an optimized adenosine deaminase harboring V82S and Q154R mutations, was developed for rice (Yan et al., 2021). TadA9 is compatible with multiple Cas9 variants, including nSpCas9, nSpCas9-NG, nScCas9, and SpRY, with near-PAM-less capability. Compared to TadA8e, TadA9 extends the editing window, enabling efficient editing of previously challenging endogenous target sites and exhibiting robust editing efficiency in commercial rice cultivars (Yan et al., 2021).

Cytosine base editor (CBE), termed Td-CBEs or TadCBEs, was recently developed through two primary methodological approaches: strategic re-engineering and phage-assisted continuous evolution of the adenine deaminase TadA-8e, targeting efficient and specific CRISPR-based cytosine base editing (Neugebauer et al., 2022; Chen et al., 2022). The introduction of an N46L mutation in TadA-8e strategically ablated its inherent adenine deaminase activity. By systematically fusing a diverse array of TadA-8e mutants with uracil glycosylase inhibitors (UGIs), researchers generated multiple Td-CBEs demonstrating either comparable activity to CBE4max or enhanced accuracy for C: G to T: A base editing (Chen et al., 2022).

Concurrently, through phage-assisted continuous evolution, an optimized TadA8e capable of cytidine deamination was successfully engineered (Neugebauer et al., 2022). This evolved TadA cytidine deaminase variant incorporates mutations within DNA-binding residues, substantially modifying enzyme selectivity to preferentially catalyze deoxycytidine deamination over deoxyadenosine. Relative to conventional CBEs, TadA-derived cytosine base editors (TadCBEs) demonstrate comparable or superior on-target activity, a more compact molecular architecture, and significantly reduced Cas-independent DNA and RNA off-target interactions (Neugebauer et al., 2022).

### Prime editors

Prime editing (PE) provides significant improvements in genome editing by enabling precise DNA modifications—including targeted insertions, deletions, and substitutions—without causing double-strand breaks in plants (Kim et al., 2023; Perroud et al., 2023; Xu Y. et al., 2023). The PE system consists of two main components: a prime editor that combines a catalytically impaired Cas9 nickase with a reverse transcriptase and a prime editing guide RNA (pegRNA) that directs the editing to a specific genomic location while encoding the desired genetic modification (Mikhaylova et al., 2024; Volodina et al., 2024; Zhang W. et al., 2024; Liu et al., 2023). This unique combination facilitates the installation of all 12 types of point mutations and small indels without the need for donor DNA templates (Liu J. et al., 2022).

PE offers several advantages over CRISPR/Cas9 and other genome editing techniques, including higher precision, reduced off-target effects, and independence from the cell’s repair mechanisms, which minimizes unintended mutations (Volodina et al., 2024; Zeng et al., 2024; Xu Z. et al., 2023). Recent studies have demonstrated a significant increase in PE efficiency, with variants like PE6c achieving over threefold increases in editing efficiency in rice (Cao et al., 2024). Additionally, incorporating T5 exonuclease into the PE system has resulted in up to 2.9-fold increases in editing efficiency across various plant cells (Liang et al., 2023), while integrating cellular factors, such as the small RNA-binding protein La, has further enhanced efficiency (Yan et al., 2024). Engineered pegRNAs (epegRNAs) have also shown improved activity and precision in editing essential plant genes (Salem et al., 2023). Despite its promise, several challenges remain in developing and applying PE technology, including the delivery of large PE components, the need for further optimization across diverse organisms, and improving efficiency and specificity in various plant species (Hosseini et al., 2024; Zeng et al., 2024; Huang and Liu, 2023).

PE empowers the rapid development of novel traits in agriculturally important crops by enabling precise genome modifications without donor DNA (Mikhaylova et al., 2024). It holds promise for enhancing traits such as yield, stress resistance, and nutritional content, which are critical for food security amid climate change (et al., 2024). Successful applications include rice, where conditional knockdown of *OsMLH1* has improved PE systems while maintaining fertility (Liu X. et al., 2024). In wheat, the development of ePPEplus has significantly boosted editing efficiency by 33-fold, allowing multiplex editing of up to eight genes (Ni et al., 2023). Moreover, an enhanced prime editing methodology in *Physcomitrium patens* has enabled routine editing, showcasing the potential for gene modification through direct selection (Perroud et al., 2023).

Ongoing research and development are essential to address these challenges and fully realize the potential of prime editing in crop improvement and genetic research (Li J. et al., 2022; Huang and Liu, 2023). As scientists continue to refine and expand PE capabilities, it is set to play an increasingly important role in advancing agricultural biotechnology and tackling global food security challenges (Ma et al., 2018).

### New emerging gene editing technologies

Gene editing technologies have revolutionized the field of biotechnology, offering precise tools for modifying genetic material. Among these technologies, Fanzor represents a novel class of RNA-guided DNA endonucleases that have been identified in eukaryotes and their viruses. Fanzors are homologous to the prokaryotic TnpB proteins and have been detected in various eukaryotic genomes, suggesting a widespread presence beyond prokaryotic systems (Jiang et al., 2023). These enzymes are characterized by their ability to be programmed by RNA to target specific DNA sequences, making them a promising tool for genome editing applications in eukaryotic cells (Jiang et al., 2023). Fanzors function as RNA-programmable DNA endonucleases, similar to the well-known CRISPR/Cas systems. They possess a rearranged catalytic site within the RuvC domain, which is crucial for their endonuclease activity. Unlike some other nucleases, Fanzors lack collateral cleavage activity, which can be advantageous for precise genome editing (Jiang et al., 2023). The evolutionary analysis of Fanzors indicates that they have adapted extensively to function in eukaryotic cells, acquiring features such as introns and nuclear localization signals (Jiang et al., 2023). This adaptation suggests a long-term evolutionary process that has enabled Fanzors to integrate effectively into eukaryotic cellular machinery. Fanzors are derived from a unique lineage of bacterial enzymes, specifically the IS607 TnpBs, which have evolved into two distinct types in eukaryotes: Fanzor1s and Fanzor2s (Yoon et al., 2023; Marraffini and Sontheimer, 2010). This evolutionary pathway highlights the transition from prokaryotic to eukaryotic systems, with Fanzors co-evolving alongside their associated transposases (Yoon et al., 2023). The ability of Fanzors to be harnessed for genome editing in human cells underscores their potential as a versatile tool in biotechnology, offering new possibilities for genetic research and therapeutic applications (Jiang et al., 2023). Fanzor represents a significant advancement in gene editing technology, with its unique RNA-guided mechanism and evolutionary adaptation making it a promising candidate for future biotechnological innovations.

Gene editing using TnpB, a transposon-associated protein, represents a novel approach in the field of genome editing. TnpB is an RNA-guided DNA endonuclease that has been identified as a potential precursor to the well-known CRISPR/Cas systems, specifically Cas12 nucleases. This discovery has opened new avenues for biotechnological applications and genome editing techniques. RNA-Guided DNA Cleavage. TnpB functions as an RNA-guided endonuclease, similar to CRISPR/Cas systems. It utilizes a guide RNA, derived from its own mRNA, to direct the cleavage of DNA at specific sites. This RNA, known as omegaRNA (ωRNA), is processed by TnpB itself, enabling it to target and cleave DNA in a sequence-specific manner (Karvelis et al., 2021; Nety et al., 2023). Transposon-Associated Motif (TAM): The DNA cleavage by TnpB occurs adjacent to a specific sequence known as the transposon-associated motif (TAM). For instance, TnpB from *Deinococcus radiodurans* targets the 5′TTGAT motif, while TnpB from Sulfolobus islandicus targets the 5′TA motif. This specificity allows TnpB to generate double-stranded DNA breaks, which are crucial for genome editing applications (Karvelis et al., 2021; Xu Y. et al., 2023). Reprogrammability and Evolutionary Significance: TnpB can be reprogrammed to target different DNA sequences, making it a versatile tool for genome editing. This reprogrammability is akin to the flexibility seen in CRISPR systems, and it highlights the evolutionary link between TnpB and CRISPR/Cas nucleases. TnpB’s evolutionary journey from a transposon-encoded protein to a potential genome editing tool underscores its functional and evolutionary flexibility (Altae-Tran et al., 2023; Altae-Tran et al., 2021). Biotechnological Potential: The discovery of TnpB’s RNA-guided nuclease activity expands the toolkit available for genome editing, particularly in organisms where traditional CRISPR systems may not be as effective. TnpB’s ability to function across a range of temperatures and its presence in diverse organisms, including archaea, further enhance its potential for biotechnological applications (Xu Z. et al., 2023; Altae-Tran et al., 2021). TnpB represents a promising new system for genome editing, with its RNA-guided DNA cleavage mechanism offering a novel approach that complements existing CRISPR technologies. Its evolutionary connection to CRISPR/Cas systems and its re-programmability make it a valuable addition to the field of genetic engineering.

CRISPR-associated transposases (CASTs) represent a novel approach in genome engineering, leveraging the RNA-guided DNA binding capabilities of CRISPR systems to facilitate the insertion of large genetic payloads without the need for DNA double-strand breaks. This method offers a promising alternative to traditional genome editing techniques, such as nuclease-based and prime editing approaches, due to its potential for high efficiency and programmability (George et al., 2023a; Walker et al., 2023a; George et al., 2023a). CASTs utilize nuclease-deficient CRISPR effectors to direct the integration of DNA at specific target sites. This process is primarily guided by RNA, which ensures the precise insertion of genetic material. The Type V-K CAST system, for instance, employs Cas12k to achieve accurate target selection, thereby facilitating RNA-dependent transposition (George et al., 2023a; George et al., 2023b). In addition to the RNA-guided mechanism, Type V-K CASTs also exhibit an RNA-independent transposition pathway. This untargeted integration is primarily driven by the availability of TnsC filaments, which preferentially bind to AT-rich sites. The TnsB transposase further refines the specificity of the insertion site by recognizing specific sequence motifs (George et al., 2023a; George et al., 2023b). The transposition process involves complex protein-protein and protein-DNA interactions. Key components such as TnsB, TnsC, and TniQ form a transpososome complex that facilitates the integration of transposons. The Type I-F *Vibrio cholerae* CAST system, for example, requires the integration host factor (IHF) for efficient transposition, highlighting the importance of cellular factors in the assembly of the transpososome (Walker et al., 2023a; Walker et al., 2023b). CRISPR-guided transposons offer a versatile and efficient tool for genome engineering, with the ability to insert large genetic payloads accurately. The dual pathways of RNA-guided and RNA-independent transposition provide flexibility in target site selection, while the understanding of protein interactions and sequence requirements enhances the precision of these systems. As research progresses, CAST systems hold the potential to revolutionize genome editing applications across various fields (Guan et al., 2013).

### Key negative regulators in climate resilience

Negative regulator genes help plants cope with abiotic stresses like drought and salinity, hence playing a crucial part in stress responses and climate resilience. Somenegative regulators often encode transcription factors that inhibit stress-responsive pathways, fine-tuning physiological responses (Yang Z. et al., 2024; Sun et al., 2008). For example, ARR1, ARR10, and *ARR12*act as negative regulators and are critical in modulating drought responses, suggesting that targeting these genes through gene editing could enhance climate resilience (Nguyen et al., 2017).

In rice, *OsWRKY12* functions as a negative regulator by repressing genes involved in abscisic acid (ABA) signaling and secondary cell wall biosynthesis, thereby decreasing drought tolerance (Jia et al., 2024). Similarly, *PgRAV-04* in pearl millet negatively impacts drought tolerance by increasing sensitivity in transgenic plants (Wang Y.-H. et al., 2024). In soybean, *GmPRR3b* suppresses the expression of *GmABF3*, a key player in the ABA signaling pathway, affecting drought response (Li et al., 2024). While studies on *TaWRKY* genes in wheat suggest their involvement in drought stress response, specific negative regulators remain to be conclusively identified (Shakam et al., 2024). In cotton, *GhVIM28* acts as a negative regulator under salt stress, indicating a potential role in drought tolerance as well (Yang X. et al., 2024). Additionally, *GhDi19-3* and *GhDi19-4* help reduce sensitivity to salt stress by regulating reactive oxygen species (ROS) levels and are involved in calcium and ABA signaling pathways (Zhao et al., 2022a). The GhRR7 gene negatively regulates drought stress responses through its role in reactive oxygen removal systems (Zhao et al., 2022b). The *miR394* pathway, targeting F-Box proteins *ZmLCR1* and *ZmLCR2*, is linked to drought tolerance, as mutants in these genes show improved drought survival, indicating their role as negative regulators (Miskevish et al., 2023). Furthermore, drought stress represses *miR166*, leading to the upregulation of its target gene, *ATHB14-LIKE*, which enhances drought tolerance. This feedback mechanism highlights *miR166’s* role as a negative regulator in drought response (Ni et al., 2023). The *ZmGA20ox3* gene demonstrated as prominent negative regulator for enhancing drought tolerance in maize seedling, reduces Anthesis-Silking Interval (ASI) delay and decreasing the yield loss significantly in the field under drought conditions (Liu Y. et al., 2024).

These findings illustrate the complex interplay of negative regulators in enhancing drought tolerance across different crops. However, the potential negative impact of these genes on yield under non-stress conditions warrants further investigation.

Various negative regulator genes modulating stress responses influence horticultural crops’ drought tolerance. For example, the *NtAITR* family of ABA-induced transcription repressors in tobacco negatively regulates drought tolerance. CRISPR/Cas9 editing of *NtAITRs* has enhanced drought tolerance, suggesting their role in repressing ABA signaling pathways (Li M. et al., 2022). In tomato, *SlWRKY6*, while primarily a positive regulator, can interact with other *WRKY* proteins to exhibit negative regulation under certain conditions, affecting drought response mechanisms (Chen et al., 2024). In rose, *RcPP2C24* has been identified as a negative regulator that reduces drought tolerance by promoting stomatal opening, leading to increased water loss during drought conditions (Shen et al., 2024).

While *bZIP* transcription factors are generally associated with positive regulation, some *bZIP* factors can act as negative regulators under specific conditions, influencing drought stress responses through complex interactions with other signaling pathways (Tao et al., 2022). These findings underscore the intricate balance of gene regulation in plant responses to drought, where negative regulators are crucial for modulating stress tolerance mechanisms. However, targeting these genes for crop improvement poses a complex challenge, as their functions can vary significantly across different species and environmental contexts.

### Key positive regulators in climate resilience

Positive regulator genes are essential for orchestrating plant responses to environmental stressors, significantly enhancing resilience and phenotypic plasticity (Hu et al., 2023). These genes modulate various physiological processes, including stress responses, growth regulation, and defense mechanisms (Table 1). A notable example is the *ERECTA* gene family, which plays a critical role in drought tolerance by influencing root system architecture and transpiration efficiency in both *Arabidopsis thaliana* and economically important crops like Oryza sativa (Kulkarni et al., 2017; Wen J. et al., 2019). Additionally, transcription factors such as *MYB37* and *CaAIEF1* have been shown to enhance ABA sensitivity and drought tolerance in Arabidopsis and Capsicum annuum, respectively, highlighting their importance in stress response pathways (Yu C. et al., 2015; Hong et al., 2017; Cheng et al., 2022). Plant glutathione S-transferases (GSTs), glycinebetaine aldehyde dehydrogenase (BADH), choline monooxygenase (CMO) and flavanone-3-hydroxylase (F3H) were involved in the protecting plants against diverse abiotic and biotic stresses (Fan, 2024). The potential target genes like Na+/H+ antiporter *viz.*, *RtNHX1*, potassium transporter gene viz.*, RtHKT1* and Group II WRKY transcription factor viz.*, RtWRKY23* from recretohalophyte *R. trigyna* demonstrated prominent sources for abiotic stress tolerance in Arabidopsis (Fan, 2024).

**TABLE 1:** Examples of positive regulator genes for climate resilience.

Sr No.	Gene	Trait	Associated stress	References
1	CBP60g and SARD1	Encode master transcription factors that are rate-Limiting for immune resilience to warming conditions	Negative regulator of Plant immunity; Plant defense responses	Rossi (2023)
2	GAI	Regulates plant growth, particularly in response to gibberellins	Regulate plant growth and development in response to environmental change	Rossi (2023)
3	PRR5	The Pseudo response regulator 5 gene controls the circadian rhythm of plants and influences early flowering	Regulating various plant physiological processes, such as photomorphogenesis, maintenance of mitochondrial homeostasis, stress responses, and flowering time regulation	Dong et al. (2021)
4	MYB37	Overexpression of this transcription factor enhances ABA sensitivity and improves drought tolerance in *Arabidopsis thaliana*	Drought stress tolerance	Yu et al. (2015b)
5	GmTCF1a	The soybean RCC1 family gene GmTCF1a enhances plant cold tolerance, demonstrating its role as a positive regulator in cold stress responses	Cold stress	Dong et al. (2021)
6	AREB1 and RD29A	Genes are positively regulated under drought stress conditions through CRISPR/dCas9 fusion with a Histone AcetylTransferase	Drought stress tolerance	Paixão (2019)
7	R2R3 type MYB transcription factor	Involved in the cold regulation of CBF genes, leading to enhanced freezing tolerance in plants	Cold Regulation and Freezing Tolerance	Agarwal et al. (2006)
8	NMR19-4	A DNA methylation variant region associated with climate adaptation and betulin biosynthesis in birch	Drought and salt stresses	Wang et al. (2021c)

In the context of thermotolerance, the *Heat Shock Factor A1 (HsfA1)* family proteins serve as master regulators of the heat stress response (Liu Y. et al., 2022), orchestrating a complex transcriptional cascade that enhances plant resilience to elevated temperatures (Mishra et al., 2002). Research in *Solanum lycopersicum* and *Zea mays* has highlighted the critical roles of *HsfA1* and related genes *(ZmHsf05, ZmHsf12)* in activating heat stress-inducible genes and heat shock proteins, thereby improving thermotolerance (Guo et al., 2020). Molecular mechanisms underlying salt tolerance involve a diverse array of genes, including ion transporters *(OsCIPK9, TaHKT9)*, transcription factors *(WRKY75, BnaABF2)*, and genes involved in reactive oxygen species (ROS) scavenging *(PtGSTF1)*, all contributing to salinity stress adaptation (Zhou et al., 2023; Du et al., 2023; Ishka and Julkowska, 2023; Ishka et al., 2024; Zhao et al., 2016; Gao et al., 2022; Li J. et al., 2022). Additionally, genes such as *AtHDA19* and *ATILL6* regulate hormonal signaling cascades and defense mechanisms, enhancing plant immunity against pathogens and abiotic stresses (Wang L. et al., 2020; Wang Z. et al., 2020; Khan et al., 2021).

An enhancement of drought tolerance is reported through CRISPR/Cas9 editing of the *TaDREB3* gene in *Triticum aestivum* (Kim et al., 2018) and knocking out the *OsbZIP46* gene in Oryza sativa (Tang J. et al., 2019). Improved thermotolerance has been achieved by modifying the *HsfA1* gene in *S. lycopersicum*, which upregulates heat stress-inducible genes and heat shock proteins (Mishra et al., 2002). Additionally, editing genes involved in ion homeostasis and ROS scavenging has significantly improved salt tolerance in various crops (Leawtrakun et al., 2024; Wani et al., 2020). Targeted modification of the *ERECTA* gene in *A. thaliana* has also enhanced drought tolerance by optimizing root architecture and transpiration efficiency (Wen W. et al., 2019; Lim et al., 2018). ABA-stimulated Calcium-dependent protein kinases (CDPKs) from grape berry, i.e., ACPK1 is involved in ABA signal transduction as a positive regulator (Yu et al., 2007).

Epigenetic mechanisms are crucial in regulating gene expression and stress responses in plants. Modifications like DNA methylation and histone changes significantly enhance stress responses in leguminous crops, such as *Cicer arietinum* (Chandana et al., 2022). The chromatin’s plasticity during environmental changes suggests that chromatin regulators and associated enzymes could be key targets for epigenetic engineering to improve climate resilience (Kumar, 2017; Kumar, 2018). Recent studies have also underscored the importance of small RNAs and the plant microbiome in boosting climate resilience. Non-coding RNAs (ncRNAs) are vital for regulating gene expression and stress responses, contributing to sustainable yields under climate change (Yadav et al., 2024). Additionally, the plant-associated microbiome enhances growth, fitness, and resistance to climate-induced stresses, highlighting the role of microbial interactions in strengthening plant resilience (Wahdan et al., 2021).

### Novel trait development for climate resilience in crops

Recent studies highlight the potential of gene editing techniques, such as CRISPR/Cas9, to modify key genetic traits associated with drought resistance in various crops. One promising application is the manipulation of gibberellin synthesis pathways, which are crucial for plant growth during drought conditions. Liu Y. et al. (2024) demonstrated that editing the *ZmGA20ox3* gene in maize improved plant architecture and enhanced drought tolerance. This suggests that targeted modifications in hormone regulation can significantly bolster a plant’s ability to withstand water scarcity. Furthermore, advancements in genomics-assisted breeding have enabled researchers to identify drought-related genes in crop wild relatives. These genes can be incorporated into modern cultivars to improve their drought resistance (Kapoor et al., 2021). The integration of stay-green traits, which prolong photosynthetic activity during drought, is another critical area of focus. Research has identified potential targets within the PIN-FORMED gene family that could be manipulated to develop stay-green cereal varieties, ensuring yield stability under drought conditions (Wong et al., 2023). Research on chickpeas has demonstrated that targeting specific genes can improve drought tolerance, contributing to developing more resilient leguminous crops (Roy and Sandhu, 2024). Similarly, studies on sorghum have highlighted genome editing’s potential to enhance stress tolerance, which is vital for sustaining food security amid climate change (Parikh et al., 2021).

Moreover, a comprehensive approach that integrates multi-omics strategies—combining genomics, proteomics, and phenomics—has been emphasized to improve the efficiency of breeding programs aimed at generating climate-resilient crops. This integration allows for a better understanding of plant responses to abiotic stresses and facilitates the identification of novel genetic targets for editing (Ndlovu, 2020; Shahzad et al., 2021). Such advanced biotechnological interventions are increasingly essential for developing sustainable agricultural practices that can mitigate drought stress (Şimşek et al., 2024).

#### Gene editing for drought and heat tolerance

Water use efficiency (WUE) is another trait that can help design resilient crops. For instance, studies have shown that manipulating genes involved in root growth, such as *TaDREB2* and *TaERF3* in wheat, can significantly improve drought resilience (Kim et al., 2017). In grapevines, regulating stomatal density by editing genes like *VvEPFL9-1* has resulted in improved water conservation and higher WUE under drought conditions (Clemens et al., 2022). Additionally, Song et al. (2023) proposed a model for the evolution of stomatal regulators in C3 and C4 crops, highlighting opportunities for enhancing drought tolerance through genetic modification. Manipulating stress response pathways has also shown great promise. For example, knocking out the *AITR* gene in Arabidopsis enhanced both drought and salinity tolerance (Chen et al., 2021). Targeting ABA signaling genes, such as PYL receptors, has improved drought tolerance across various species (Kumar, 2023). Multi-omics approaches have identified targets like *OsBADH2* and *OsMPK2* in rice that could enhance drought resilience (Kumar, 2023). In leguminous crops, successful editing of the *4CL* and *RVE7* genes in chickpea protoplasts has demonstrated potential for improving drought tolerance (Badhan et al., 2021). Additionally, engineering trehalose metabolism in Arabidopsis, where a CRISPR-edited line mimicked the substrate binding site of the trehalase enzyme, resulted in increased drought tolerance (Mahalingam et al., 2022). This case illustrates the potential of gene editing to modify metabolic pathways, enhancing stress tolerance and opening avenues for similar approaches in other cultivated crops. In addition to metabolic engineering, manipulating root architecture has emerged as a key focus in drought tolerance research. For instance, Nascimento et al. (2023) explored genes that promote root development, enhanced root growth, and improved water uptake, demonstrating their potential as targets for CRISPR/Cas9 gene editing. Their findings highlighted how knocking out certain genes negatively impacts root development under drought conditions, ultimately enhancing overall crop resilience to water scarcity.

Manipulating genes involved in the ABA signaling pathway through gene editing has shown promising results in enhancing drought tolerance. Furthermore, the application of gene editing techniques extends beyond model plants; for example, targeting the *GhCLA1* gene in cotton has been shown to improve drought tolerance (Gao et al., 2017). This study not only confirmed the efficacy of gene editing in a major crop but also paved the way for future applications aimed at enhancing drought resilience in economically important species across various crops.

Heat stress poses a significant threat to agricultural productivity; here, we focuson case studies in heat shock response, photosynthesis, and membrane stability. Heat shock proteins (HSPs) protect cellular functions under thermal stress. Several HSP-encoding genes, including *HSP70*, have been identified as crucial for heat tolerance in various cereal crops (Baldoni et al., 2021). The expression of these genes has been enhanced through gene editing techniques, successfully improving survival rates under high-temperature stress in crops like wheat and maize. Additionally, the transcription factor *HsfA2* has been discovered; its overexpression promotes the expression of downstream heat-responsive genes, making it essential for developing heat stress tolerance (Xie et al., 2023; Xie et al., 2021).

Photosynthesis, another critical physiological process affected by heat stress, has also been the focus of research. One study demonstrated that grafting cucumber onto heat-tolerant rootstocks significantly reduced heat-induced photosynthesis (Xu et al., 2018). Proteomic analysis revealed that key enzymes involved in photosynthesis were upregulated in the grafted plants, showcasing the potential for gene editing to enhance photosynthetic efficiency and productivity under elevated temperatures.

Membrane stability is crucial for maintaining cellular integrity during heat stress. Research has highlighted the role of ethylene-responsive transcription factors in regulating membrane stability in cotton under high-temperature stress (Liang et al., 2021). Moreover, Zha et al. (2023) described how overexpressing genes such as *VvDREB2c*, *HSP70,* and *HsfA2* improve heat tolerance in Arabidopsis. The role of salicylic acid in heat tolerance has also been explored through gene editing. Researchers demonstrated that exogenous application of salicylic acid could enhance heat tolerance in waxy maize by modulating the expression of heat shock response genes (Guo et al., 2022).

**FIGURE 3:**
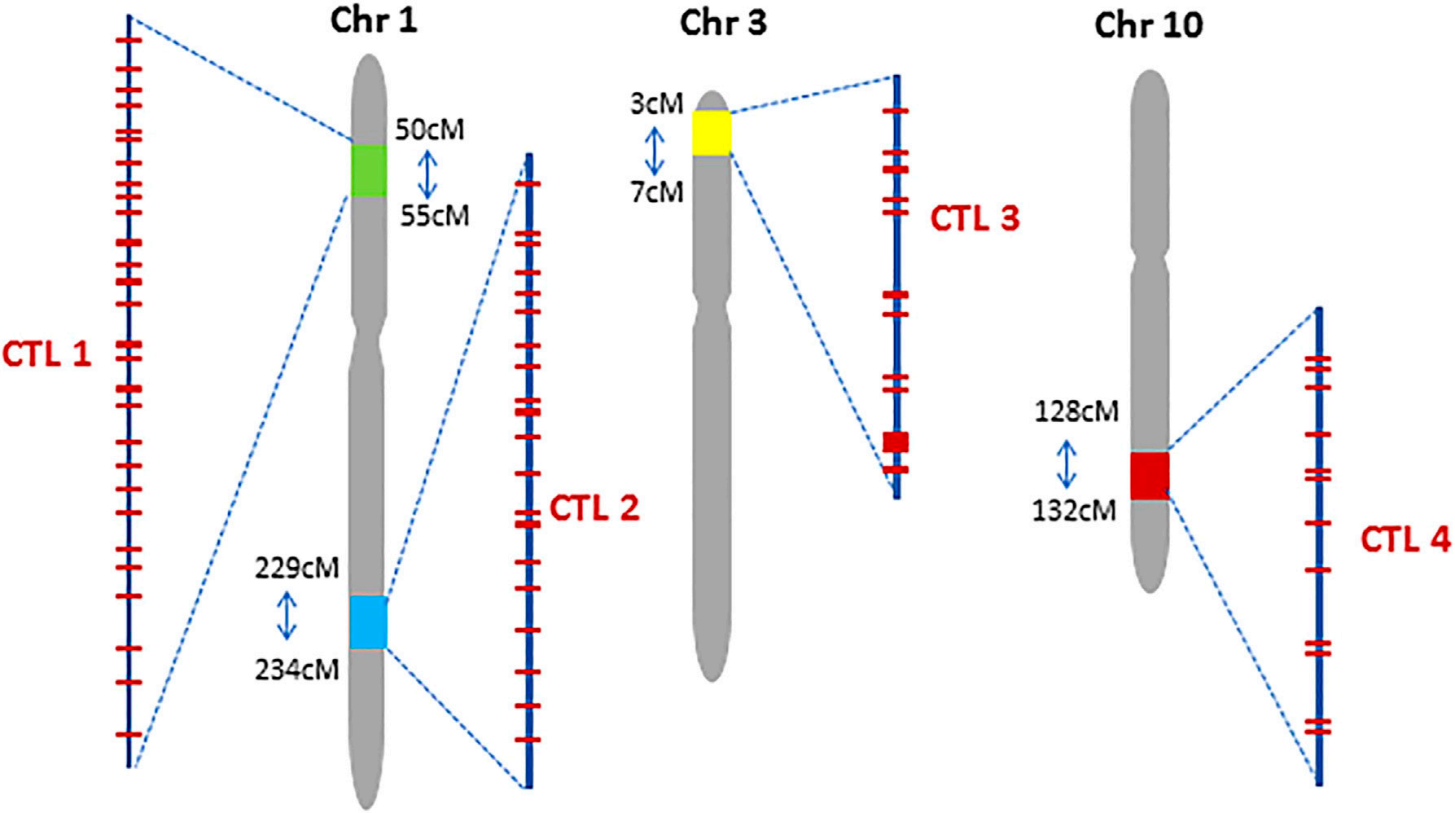
Chromosomal location of four Complex Trait Loci (CTL) in the maize genome. Red bars within each CTL represent preselected CRISPR targeting sites (Figure from Gao et al., 2022; Copyright: CC BY License).

Another example isthat engineering *SlHyPRP1* protein domains in tomatoes has improved multi-stress tolerance, including heat stress. Genome-edited tomato lines have demonstrated better germination and vegetative growth under heat-stress conditions, showcasing the potential of these edited genes to enhance heat resilience (Tran et al., 2023). Moreover, a notable case involves the overexpression of the heat shock factor *TaHsfA6f* in wheat, which resulted in increased ABA levels, thereby improving tolerance to multiple abiotic stresses, including heat (Bi et al., 2020). In rice, researchers have exploited quantitative trait loci (QTLs) associated with heat stress tolerance, targeting candidate genes related to heat shock proteins (HSPs) and calmodulin-binding proteins to enhance heat tolerance (Kilasi et al., 2018). Moreover, in Maize, the Complex Trait Loci (CTL, Figure 3) have been used for novel trait development. The role of ABA in mediating heat stress tolerance has also been explored. ABA is critical in preventing pollen abortion under high-temperature stress in rice spikelets, and manipulating ABA signaling pathways through gene editing could enhance heat tolerance during critical reproductive stages (Rezaul et al., 2018). Additionally, research has highlighted the proactive role of heat shock factor C2a in wheat, which operates through an ABA-mediated regulatory pathway to protect developing grains from heat stress (Hu et al., 2017). By enhancing the expression of this transcription factor using CRISPR/Cas9, researchers can potentially improve heat tolerance in wheat during crucial growth stages, thus safeguarding yields in hot tropical climates.

#### Gene editing for salt tolerance

By stacking multiple edited genes, researchers can greatly improve crop resilience to various stressors, such as drought, salinity, and heat. This approach synthesizes findings from numerous studies that focus on integrating multiple traits for robust climate resilience. The combined editing of genes to enhance stress tolerance offers a promising strategy for developing crops that can withstand challenging environmental conditions.

Salt stress is a major abiotic challenge that significantly impacts agricultural productivity, making the enhancement of salt tolerance in crops essential for ensuring food security in saline areas. Plants manage salt stress through mechanisms that regulate ion homeostasis, particularly involving sodium (Na^+^) and potassium (K^+^) transporters. One study highlighted that using CRISPR/Cas9 to mutate *GmAIT* genes in soybeans improved salinity tolerance (Wang T. et al., 2021). The *AITR* transcription factors play a key role in controlling ion transport and homeostasis. By knocking out these factors, researchers observed improved K^+^/Na^+^ ratios in soybean plants, which are crucial for maintaining cellular function during salt stress. This example demonstrates how precise gene editing can directly modify ion transport pathways to enhance salt tolerance in crops.

In rice, the *OsCIPK9* gene has been identified as a crucial regulator of sodium ion homeostasis. It interacts with *OsSOS3*, affecting salt-related transport and improving salt tolerance (Zhou et al., 2023). Editing the genomic region of *OsCIPK9* could create new alleles that enhance the plant’s ability to manage sodium levels during salt stress. Osmotic adjustments are another critical aspect of salt tolerance. The CRISPR/Cas9, gene editing approach, can enhance the expression of specific transporters involved in osmotic regulation, thereby improving the plant’s ability to maintain turgor pressure and cellular integrity under saline conditions. This strategy is particularly relevant for crops grown in saline soils, where osmotic stress can severely limit growth and yield. Furthermore, the integration of multi-omics approaches, such as comparative transcriptomic analyses, has helped identify essential genes associated with salt stress response in sorghum (Jeon et al., 2023). These insights can be exploited to edit targets that enhance salt tolerance through various mechanisms, including ion homeostasis, osmotic adjustments, and oxidative stress management (Yuan et al., 2024).

Oxidative stress response is also vital for plant adaptation to salt stress. MYB transcription factors regulate ion homeostasis and control reactive oxygen species (ROS) levels during salt stress (Ahmad et al., 2023). Editing these transcription factors with CRISPR/Cas9 can accelerate a plant’s ability to manage oxidative stress, thereby improving overall salt tolerance. Additionally, a study reviewed various transgenic approaches to improve crop salt tolerance, emphasizing the importance of pyramiding multiple genes involved in salt stress responses (Hsu et al., 2020; Kotula et al., 2020; Liu et al., 2021). By combining gene editing strategies with traditional breeding methods, researchers can develop crops with enhanced resilience to salinity, ultimately leading to improved agricultural productivity in saline environments. Understanding the genetic basis of salt tolerance in halophytes is also necessary. Researchers identified some target genes in glycophytes (Grigore and Vicente, 2023) and could exploit opportunities through gene editing to confer resilience to salinity.

One prominent example is the study focusing on the *GhA08G1120 (GH3.5)* gene in cotton. Researchers demonstrated that suppressing this gene resulted in enhanced drought and salt stress tolerance (Kirungu et al., 2019). The *GH3.5* gene is involved in auxin metabolism, and its manipulation led to increased proline accumulation, which plays a crucial role in osmotic adjustment and the detoxification of reactive oxygen species (ROS). The *OsMYB6* gene has been identified as a key regulator of abiotic stress responses in rice. Overexpressing *OsMYB6* in transgenic rice improved resistance to drought and salinity stress (Tang Y. et al., 2019). This study highlighted several abiotic stress-related genes, including *OsLEA3* and *OsDREB2A*, known to enhance stress tolerance. These examples underscore the potential of targeting transcription factors through gene editing to improve salt tolerance in crops.

The *GmST1* gene in soybeans has also shown promise; it reduces ROS production and enhances sensitivity to ABA during salt stress. Overexpressing *GmST1* in Arabidopsis improved drought tolerance and reduced water loss from leaves (Ren et al., 2016; Ma et al., 2021). Additionally, the role of auxin in salt tolerance has been explored through the *CqEXPA50* gene identified in quinoa. Manipulating this gene, which is involved in auxin-mediated responses, enhances salt tolerance by improving cell expansion and osmotic adjustment (Sun et al., 2022; Zhang et al., 2021).

Targeting multiple genes simultaneously facilitates the pyramiding of traits, including enhanced root architecture, improved water use efficiency, and increased resistance to pests and diseases. This approach accelerates breeding and increases the likelihood of developing crops that can thrive under changing climatic conditions. The importance of root traits in enhancing crop resilience has also gained attention. Modulating root hair development can optimize nutrient and water uptake, thereby improving crop yield and resilience (Tsang et al., 2023). By targeting key genes that control root hair development through gene editing, breeders can enhance crops’ ability to access water and nutrients in challenging environments, further contributing to climate resilience. In addition to root traits, stacking traits related to osmotic adjustment and oxidative stress response are essential for developing resilient crops. Research is exploring the potential of multiplex-CRISPR gene editing constructs to accelerate genetic gains in underutilized crops, focusing on traits that enhance tolerance to abiotic stresses (Sharma et al., 2022).

### Recent advancements in genome editing

The CRISPR variants Cas12 and Cas13 offered improvements in specificity and reduced off-target effects compared to Cas9 and proved to be a highly efficient gene editing tool with complementary properties and functionality to Cas9 (Zetsche et al., 2017; Zhang et al., 2017). The Cas12a does not require a tracrRNA for activation and is potentially exploited for multiplexed editing (Martin et al., 2024). Additionally, Cas12a′s trans-nuclease activity is utilized to detect sequence-specific nucleic acid (Chen et al., 2018). In contrast, Cas13 is an RNA-targeting enzyme useful for modifying gene expression at the transcript level.

The first-generation CRISPR-based gene editing tools face several challenges, including specificity, targeting scope, dependency on endogenous DSB repair mechanisms, absence of efficient delivery methods, lack of effective vectors and rigidity of cell or organisms, off-target activity, etc. (Kadam et al., 2018; Martin et al., 2024). Also, the requirement of a specific PAM Sequence for targeted genome editing is crucial, and even though it reduces off-target activity, it often results in suboptimal DNA cleavage, editing efficiencies (Ran et al., 2015; Edraki et al., 2019) and restricting the scope of targeted edition. Recent advancements in gene-editing technologies have led to the development of artificial Cas9 variants with relaxed PAM specificities, which improve efficiency but are associated with an increase in off-target effects and a decrease in target specificity (Ran et al., 2015). The control as well as stimulating, as well as enhancing efficiency of HDR within a coRanntrolled editing system ruling out adverse editing outcomes, especially large deletions, chromosomal rearrangements, and even chromosome loss (Kosicki et al., 2018; Cullot et al., 2019; Leibowitz et al., 2021).,Enhancement of HDR methods pertains touse of asymmetric *ssODN* templates (Richardson et al., 2016), Silent mutations introduction to obstruct recurrent cleavage at the target site (Paquet et al., 2016), tethering of the repair donor template to the break site (Carlson-Stevermer et al., 2017),and manipulation of the cell cycle combined with the delivery of pre-assembled Cas9-ribonucleoparticles (Lin et al., 2014).

The delivery of existing Cas9 or Cas12a enzymes and their guide RNAs are available in different methods, among which electroporation (nucleofection) or liposome-mediated transfection remain the methods of choice, and it is delivered in the form of RNA, plasmid DNA, or in ribonucleoprotein (RNP)complexes. The delivery of the enzymes expressed the limitations of less efficiency, tissue-specific, immunogenicity, and species-specificity, etc. (Chen et al., 2020; Cheng et al., 2023). The limitations of first-generation gene editing techniques have been enhanced by the latest evolved versatile new tools, including precision editing, safety concerns, and minimal unintended editing consequences. The currently available technologies provide amuch more custom-made approach to genome editing, with specificity for certain types of edits or delivery methods.

#### High-fidelity Cas9 variants

The variant SpCas9 has been developed to resolve the issue of off-target activity and improve specificity using two complementary approaches. Several other variants of Cas9 and Cas12a enzymes have been developed with improved specificity and efficiency, but their efficiency and specificity may vary depending on the different target DNA and utility (Kim et al., 2022). Nevertheless, each of the high-fidelity variants is not universally acceptable and possesses certain limitations (Martin et al., 2024).

#### Guide RNA modifications

Modifications to the guide RNA (gRNA) have been developed to reduce off-target effects, though these changes often come at the cost of editing efficiency. Various approaches have been explored, including truncated gRNA, 5′end modifications, hybrid RNA-DNA guides, and nucleotide substitutions within the gRNA sequence. Truncating the gRNA from 20 nucleotides to 17–18 nucleotides has been shown to reduce off-target activity (Fu et al., 2014), though it can also decrease editing efficiency (Sai, 2021). Modifications at the 5′end of the gRNA, such as the addition of secondary structures (Kocak et al., 2019) or unpaired nucleotides (Kulcsár et al., 2020), have also been found to lower off-target activity. The use of “hybrid” RNA-DNA guides has demonstrated significant improvements in gene editing specificity (Donohoue et al., 2021). Additionally, chemical modifications involving 2′-O-methyl or 2′-fluoro nucleotides and phosphorothioate linkages in the gRNA have proven to be effective strategies for enhancing both the specificity of Cas9 and the stability of the gRNA (Martin et al., 2024).

#### Alternative PAM genome editors

The requirement of aspecific PAM recognition site for CAS nucleases is becoming the major constraint in genome editing in several species. In this concern, to get rid of the availability of specific PAM sites, the modified variants of SpCas9 and Cas12a have been developed. The modified variants of SpCas9 and Cas12a bypass the need for specific PAM sites. Efforts to expand the PAM targeting range of SpCas9 have included structure-based rational engineering and amino acid substitutions in its PAM-interacting domain. These modifications have led to the creation of SpCas9 variants such as VQR, EQR, and VRER, which can recognize broader PAM sequences like NGAN, NGNG, and NGCG, respectively (Kleinstiver et al., 2015; Hirano et al., 2016).

Recent advancements have also focused on engineering SpCas9 variants that can target NRN PAM sequences, significantly broadening their application potential. Efforts are ongoing to develop PAM-free Cas9 molecules to eliminate PAM site constraints altogether, thereby increasing the flexibility and effectiveness of genome editingthrough structure-based rational engineering or by altering amino acid substitutions in the PAM-interacting domain (Walton et al., 2020). This could result in the development of wider-ranged VQR, EQR, and VRER SpCas9 variants, enabling the targeting of NGAN, NGNG, and NGCG PAMs, respectively (Kleinstiver et al., 2015; Hirano et al., 2016).

#### Base editing

The discovery of spontaneous hydrolytic deamination of cytosine converting C-G base pairs to T-A in humans highlighted the potential of base editing for direct base pair conversion without requiring double-strand breaks or homology repair templates (Komor, et al., 2016). Base editors consist of an inactive CRISPR/Cas9 (dCas9/Cas9 nickase) and a deaminase (cytosine or adenosine). They are classified into DNA and RNA-based editors. Current DNA base editors include cytosine base editors (CBEs) and adenine base editors (ABEs) for C-T and A-G conversions. First-generation base editors had limitations such as low efficiency and base excision repair (BER) activity that reversed edits (Komor et al., 2016). Second-generation editors, such as APOBEC-XTEN-dCas9-UGI, added an uracil DNA glycosylase inhibitor (UGI) to improve C-to-T conversion (Komor et al., 2016) but still had <0.1% indel formation, limiting precision (Azameti and Dauda, 2021).

Third-generation base editors, with rAPOBEC1 fused to the N-terminus and UGI at the C-terminus of nickase Cas9 D10A, offered higher editing efficiency and reduced off-target effects but required an NGG PAM sequence, limiting their scope (Kim et al., 2017). Fourth-generation editors (SpBE4, SaBE4) improved upon this by adding two UGI molecules to the C-terminus. The BE4-GAM variant further enhanced efficiency by fusing the Gam protein (a DNA end-binding protein) to the Cas9 nickase N-terminus.

The adenine base editor (ABE) comprises three main components: a mutant transfer RNA adenosine deaminase (TadA), sgRNA, and Cas9 nickase, facilitating A-to-G conversions (Gaudelli et al., 2017). To enhance editing efficiency and minimize off-target effects, approximately eight ABE variants have been developed. The ABE-Plant version 1 Simplified (ABE-P1S) demonstrated higher editing efficiency in rice compared to the commonly used ecTadA-ecTadA*7.10-nSpCas9 (D10A) fusion (Hua et al., 2020). In developing herbicide-resistant commercial rice, TadA9 showed compatibility with multiple nickase systems, including CRISPR/SpCas9, CRISPR/SpCas9-NG, CRISPR/SpRY, and CRISPR/ScCas9, achieving high editing efficiency across four herbicide target genes.

A dual-base editing system was created by fusing both cytidine and adenosine deaminases to Cas9, allowing simultaneous C→T and A→G substitutions using a single sgRNA within one target site (Li et al., 2020b). Addressing the limitations of ABEs and CBEs, which can only achieve up to 33% of possible base substitutions, researchers developed Transversion Base Editors to expand editing capabilities. Notably, Zhao et al. (2022a) introduced glycosylase base editors (GBEs), which enable C-to-A and C-to-G conversions.

#### PAM-less base editing

PAM-less base editing has expanded the possibilities in genome editing by providing access to previously unreachable PAM sequences. Normally, SpCas9 recognizes the ‘NGG’ PAM sequence; however, researchers have developed variants that reduce this specificity, enabling recognition of a single guanine (G) nucleotide. These include variants like xCas9-3.7, SpCas9-NG, and ScCas9 (Azameti and Dauda, 2021). Building on this, Walton et al. (2020) introduced the SpG variant, which can recognize a broader range of NGN PAMs. Further optimization led to SpRY, a variant capable of targeting nearly any PAM. Although this technology significantly broadens the targetable genome space, it has limitations. For example, SpCas9-NG shows reduced editing efficiency on 5′-NGC-3′ PAM targets and tends to increase off-target activity (Nishimasu et al., 2014).

#### Multiplex base editing systems

It provides a multifunctional CRISPR system that performs dual and tri-functional base editing but suffers from the requirement of each Cas protein specific to its own PAM sequence (Lian et al., 2017). The primer base editors CBE and ABE have been improved a lot through several modifications with increased specificity and reduced deaminase—induced off-target activity (Doman et al., 2020). Further base editing option has been expanded beyond ABE and CBE and covers A-to-C, A-to-Y and C-to-G (Martin et al., 2024) edits. Being an alternative to CRISPR/Cas nine and efficient system (Li M. et al., 2022; Veillet, et al., 2019), base editing catches the universal attraction of the scientist for functional genomics studies and in the quest of mining the important traits governing SNPs in various crop plants (Azameti and Dauda, 2021), improved crop varieties could be developed by the programmed and precise conversion of targeted single bases in the genomes of plants. It has been utilized for several applications, including single point mutations and targeted mutation induction for therapeutic corrections of diseases (Musunuru et al., 2021), mutational screening, and gene knockout among the genomes (Hanna et al., 2021; Que et al., 2010). Besides controlled editing, BEs are facing several limitations, including low efficiency, large editing windows, and off-target activity (Martin et al., 2024). Also, they possessundesired genotoxic effects by generating DSBs, deletions, and translocations at the on-target locus.

#### Synthetic gene activators

Synthetic gene activators are also a promising strategy that activates genes by tethering an autonomous transcription activation domain (TAD) to the gene promoter via a programmable DNA-binding module. Various Synthetic gene activators are available, including dCas9-TADs, zincfinger protein–TADs, and transcription activator-like effector (TALE)–TADs. Among which, the nuclease-dead *S. pyogenes* Cas9 (dCas9) protein provides simplicity and multiplexity (Didovyk et al., 2016). Further advancements in genome editing technologies have developed deactivated Cas9 (dCas9) based transcriptional activation systems (Li et al., 2017). In animal cells, the dCas9-based transcriptional activation systems, like VPR, SAM, and SunTag, are being utilized, while in plant cells, dCas9–TV system offers stronger transcriptional activation of single or multiple genes (Li et al., 2017). This method is particularly useful for correcting point mutations that cause genetic diseases or undesirable traits. For instance, base editing has improved disease resistance and enhanced nutritional profiles in crops like tomatoes and potatoes.

#### Prime editing

Prime editing is a more recent development that enables precise insertion, deletion, or replacement of DNA sequences with high accuracy. It uses a “prime editor” complex that includes a modified CRISPR protein and an engineered reverse transcriptase. This technology holds promise for fixing a broader range of genetic mutations with fewer unintended effects, potentially improving traits such as yield, pest resistance, and environmental resilience (Liu, et al., 2023). The prime editor system contains a fusion of nCas9 (H840A) and reverse transcriptase enzyme derived from Moloney murine leukemia virus (M-MLV RT), prime editing guide RNA (pegRNA)/sgRNA consisting of a reverse transcriptase template, and a primer-binding site at the 3' end of the sgRNA. It produces indels and base replacements without the limitations of specific PAM. Prime editing has been extensively utilized in rice, wheat, and maize and induced point mutations, deletions, and insertions (Lin et al., 2020; Xu et al., 2020b).

#### Epigenome editing

Epigenome editing involves modifying the epigenetic marks on DNA, such as DNA methylation or histone modification, to influence gene expression without altering the underlying DNA sequence (Kumar and Mohapatra, 2021). This approach can be used to fine-tune gene expression levels, which may help in optimizing crop traits like flowering time and stress responses. This can be achieved through fusion between nuclease-dead Cas9 with DNA and histone-modifying enzymes, which restructure the chromatin at precise loci of the genome and enable induction or repression of expression of the target gene (Nuñez et al., 2021). It can be achieved through CRISPR off as well as CRISPR on mechanism enabling silencing as well as reactivation of gene expression respectively (Nunezet al., 2021). It suffers from the challenges of binding Cas9 to off-target sites and influencing histone and chromatin modifiers, which inadvertently affect the transcription of off-target genes (Kuscu et al., 2014).

#### Bio-mimicking via promoter, allele, or gene replacement

The introduction of foreign DNA/genes into crops is treated as transgenic and often faces the challenges of lengthy regulatory concerns and consumer refusal. Such constraints can be overcome by CRISPR technology working on the principle of bio-mimicking. It offers the introduction of mutation instead of the whole gene using CRISPR, wherein the sequence of the target gene is converted into a desirable gene sequence that has a specific trait. It is being achieved through gene silencing or gene knockout via induced mutation within gene sequence and its replacement within the cultivated species, induction of mutations within allele as well as promoter region of the gene (Tan et al., 2022).

#### Emerging methods for efficient CRISPR delivery in plant systems

The advancement of plant genome editing through CRISPR technologies hinges critically on developing efficient delivery mechanisms that can overcome the inherent challenges posed by plant cellular structures. Researchers are actively exploring diverse strategies to successfully introduce CRISPR components into plant cells, addressing the complexities introduced by rigid cell walls and intricate genomic landscapes.

Non-viral delivery methods have emerged as a promising avenue for CRISPR component transmission. Innovative nanotechnology-based approaches, including inorganic nanoparticles, carbon nanotubes, liposomes, and protein- and peptide-based nanoparticles, offer significant advantages over traditional delivery techniques. These vectors demonstrate reduced immunogenic responses and lower cytotoxicity, making them particularly attractive for genetic modification strategies (Alghuthaymi et al., 2021).

Viral vector delivery, specifically virus-induced genome editing (VIGE), represents another sophisticated approach to CRISPR component introduction. By utilizing plant RNA viruses as transient delivery vectors, this method enables high-efficiency editing and facilitates the generation of DNA-free gene-edited plants. VIGE is especially valuable for achieving tissue-culture-free editing and enhancing plants’ biotic resistance mechanisms (Uranga and Daròs, 2022; Zhang C. et al., 2022).

Ribonucleoprotein (RNP) delivery has gained significant attention as a refined method for CRISPR component transmission. This approach minimizes potential risks associated with transgene integration and off-target effects, providing a safer alternative to conventional plasmid-based methodologies. Researchers have demonstrated impressive editing efficiencies using RNPs across various plant species, including rice and citrus, underscoring the method’s potential for generating transgene-free genome-edited plants (Zhang Y. et al., 2022; Zhang et al., 2019).

Traditional transformation techniques, such as Agrobacterium-mediated transformation and particle bombardment, continue to play a crucial role in CRISPR delivery. While these methods have historically been successful in a limited number of plant species and often require extensive tissue culture and regeneration procedures, ongoing research aims to enhance their capabilities. Current developments focus on achieving genotype-independent delivery and implementing DNA-free editing protocols (Laforest and Nadakuduti, 2022; Ghogare et al., 2021; González et al., 2021).

Despite considerable progress in developing novel delivery methods, significant challenges persist in establishing universally efficient and scalable CRISPR delivery mechanisms across diverse plant species. Key research priorities include improving plant regeneration from edited protoplasts and developing genotype-independent delivery technologies. Future research directions will concentrate on optimizing existing methodologies and exploring innovative approaches to enhance the precision and efficiency of CRISPR-based plant genome editing (Laforest and Nadakuduti, 2022; Nadakuduti and Enciso-Rodríguez, 2021; Erdoğan et al., 2023).

This multifaceted approach to CRISPR delivery reflects the dynamic and evolving landscape of plant genome editing, promising transformative potential for agricultural innovation and crop improvement.

#### Off-target effects in CRISPR/Cas technology and their mitigation strategies

Off-target effects are unintended genomic modifications occur when guide RNA (gRNA) binds to sequences similar to, but not identical with, the intended target, potentially resulting in undesirable DNA cleavage and mutations (Guo et al., 2023; Lopes and Prasad, 2024; Garrigues et al., 2023).

The fundamental mechanism of off-target effects stems from the gRNA’s ability to interact with non-target genomic sites sharing sequence homology. Consequentially, these interactions can generate diverse genetic alterations, including small insertions or deletions (indels), structural variations like translocations, inversions, and extensive deletions. Such modifications pose substantial risks, particularly in therapeutic applications (Lopes and Prasad, 2024; Mengstie et al., 2024). In bacterial systems, these off-target interactions can further precipitate gene silencing and cellular toxicity, underscoring the critical importance of precise molecular targeting (Rostain et al., 2023).

Researchers have recognized these challenges and developed sophisticated mitigation strategies to enhance CRISPR/Cas9’s safety and efficacy. Bioinformatics tools have been instrumental in this endeavor, employing computational approaches to optimize gRNA design, ensuring high specificity and minimizing off-target activity. These computational strategies assist researchers in strategically selecting target sites and validating experimental outcomes (Naeem and Alkhnbashi, 2023; Zhang et al., 2023).

Innovative technological approaches have further expanded the toolkit for managing off-target effects. Researchers have explored strategies such as utilizing Non-Homologous End-Joining (NHEJ)-deficient strains in organisms like filamentous fungi, which have demonstrated significant reductions in off-target mutations and enhanced genomic stability (Garrigues et al., 2023). Machine learning and deep learning models have emerged as powerful predictive tools, enabling researchers to anticipate and comprehend potential off-target activities with unprecedented precision (Vora et al., 2023).

Perhaps most promising are cutting-edge technological interventions like G-quadruplex-based CRISPR photoswitches. These innovative mechanisms provide spatiotemporal control over CRISPR activity, allowing researchers to precisely activate and deactivate the CRISPR system. Such controlled approaches substantially mitigate off-target risks by enabling more targeted genetic interventions (Deng et al., 2023).

Continuous technological advancements have been paramount in addressing these challenges. The development of high-fidelity Cas9 variants and strategically modified gRNAs represents a significant stride toward minimizing off-target effects, progressively refining the precision of gene editing technologies (Mengstie et al., 2024).

While off-target effects remain a substantial concern in CRISPR/Cas9 applications, particularly in therapeutic contexts, the multifaceted approach to mitigation demonstrates remarkable scientific ingenuity. Researchers are systematically addressing the technology’s inherent challenges by integrating computational prediction, technological innovation, and sophisticated molecular control mechanisms. These advancements not only enhance the safety and reliability of CRISPR/Cas9 but also expand its potential applications across diverse scientific and medical domains.

### Application of artificial intelligence and phenomics in genomic editing

Artificial intelligence (AI) constitutes machine-learning algorithms, for example, deep neural network (DNN), artificial neural network (ANN), random forest (RF), support vector machine (SVM), and advanced hi-tech equipment like the internet of things (IoT) (Baduge et al., 2022). It fascinates a hi-tech system that is capable of handling big data in a short time and making judgments rapidly and more accurately than humans (Xu Y. et al., 2023). It saves breeders time in data identification and processing. It accelerates advanced breeding through the use of high-throughput genomics and phenomics, leading to the breeding of next-generation resilient crops (Khan et al., 2021). Moreover, machine learning (ML) tools help in genomic prediction, genomic selection, deep learning, and predictive analysis help to increase the planning, learning, reasoning, thinking, and action-taking abilities, enabling digital breeding and developing next-generation crops (Shaw et al., 2022).

Machine learning (ML) tools arebeing proposed to predict off-target placement throughout crop genomes. It also enables the training and test the possible regression points that predict off-target or on-target specificities (Kaul et al., 2020). AI is helping to overcome phenomics bottlenecks; it manages phenotypic data through algorithms and programs that convert sensory data into phenotypic information. It helps in model development and enables the understanding ofgenotype-phenotype relationships with interaction with different environmental conditions (Nabwire et al., 2021). AI assists in plant phenomics systems by identifying plant developmental stages, plant images, categorization of the crops and weeds, scoring and images of the diseases of crops, etc. (Khan et al., 2021). Moreover, AI has been used in gene function analysis. The available AI algorithms may be utilized to predict the cleavage ability of theCRISPR system. AI tools are extensively utilized in genome exploration, including the identification of protein-coding genes, regulatory elements, cell-to-cell gene expression and location, protein-protein interaction networks, and metabolic pathways. Prediction and analysis of the genetic features of an individual are becoming possible through the DeepSEA and DeepBind models (Zhou et al., 2023). The various laboratory data point features such as GC content of gRNA, the secondary structure of sgRNA, CpG island, chromatic structure, ORF sequence, the gene expression profile of various developmental stages, available sgRNA and their distributions, sequence and protein databases with detailed descriptions can be linked to formulating AI models in gene editing.

Thus, integrated use of Artificial intelligence (AI) technology assisted with phenomics and genomics tools combined with genome editing tools offers projective modeling in precision editing, reduced off-target activity, minimizing unintended genetic alterations, and increased editing efficiency with promising outcomes (Xiang et al., 2021). It is being used in the coming future to predict and mine more powerful and efficient specific nucleases through data analysis, modeling, and computational biology approach. However, the technology has come up with its own challenges, like the availability of qualitative and variable data, suitable predictions, interpretations, efficient decision-making systems, etc.

### Prospects of multiple traits development in climate resilience

Stacking multiple traits in crops through gene editing technologies presents a promising avenue for developing comprehensive climate resilience. However, this approach also faces several challenges and considerations that must be addressed to ensure successful implementation. This response synthesizes insights from various studies on the complexities of pyramiding multiple edited genes for combined stress tolerance.

One primary challenge in stacking traits is the potential for negative interactions between different genetic modifications (Zonneveld et al., 2020). When multiple traits are introduced through gene editing, the interactions between these traits could lead to unforeseen physiological responses, potentially diminishing the overall effectiveness of the stacked traits (Malenica et al., 2021). Additionally, there is a risk of increased susceptibility to certain diseases or reduced fitness under specific environmental conditions, which poses a significant challenge in developing multi-trait crops (Panda et al., 2024; Dormatey et al., 2020).

A significant technical hurdle is the high-throughput mutant genotyping required to identify and confirm the presence of multiple edited genes in a single plant (Karunarathne et al., 2023). The complexity of plant genomes and gene interactions complicates the breeding process, as one gene’s expression can influence another’s expression, leading to unintended phenotypic outcomes (Kumar, 2023). Thus, there is a pressing need to understand the interactions between different stress response pathways to mitigate potential risks.

Another layer of complexity is the intricate nature of plant responses to environmental stresses. Plant responses to climate change can often contradict empirical expectations, making it challenging to predict how stacked traits will perform in real-world conditions (Putra et al., 2022). This complexity necessitates comprehensive modeling approaches and field trials to assess the effectiveness of stacked traits under varying environmental conditions. Additionally, the genetic background of the crop plays a critical role in the success of trait stacking, highlighting the importance of understanding the genetic architecture of the crop for successful trait pyramiding.

Considering the potential unintended consequences of a multiplex gene editing approach requires careful planning and robust solutions. While significant advances have been made in developing varieties tolerant to specific stresses, most efforts have focused on monogenic or oligogenic traits (Prohens et al., 2017). Furthermore, the socio-economic context surrounding the adoption of stacked trait varieties must be addressed, emphasizing effective communication and education about the benefits of these traits among end-user farmers.

Gene pyramiding has been effectively demonstrated in several crops, including rice, maize, and barley. Using CRISPR/Cas9 gene editing technologies, barley abiotic stress tolerance has been enhanced by targeting specific genes associated with drought and salinity resistance (Karunarathne et al., 2023; Yadav et al., 2023). Similarly, in maize and sorghum, a multi-trait improvement approach with resilience to acidic soils is ongoing (Guimarães and Magalhães, 2021). Regulatory considerations also play a critical role in deploying gene pyramiding strategies. The approval processes for genetically modified organisms (GMOs) vary significantly across countries, and the introduction of multi-gene constructs may face additional scrutiny due to concerns over environmental impact and food safety (Fan, 2024; Suza et al., 2018). This regulatory landscape can hinder the rapid adoption of innovative breeding techniques, delaying the availability of improved crop varieties to farmers.

Despite these challenges, the potential benefits of gene pyramiding for climate resilience are substantial. The pyramiding of genes associated with drought tolerance, salinity resistance, and nutrient acquisition has enhanced the overall resilience of crops like rice and maize (Pang et al., 2017; Shailani et al., 2020). Combining multiple stress tolerance traits allows a researcher to develop crop varieties that survive and thrive under adverse conditions. This comprehensive approach improves individual plant performance and contributes to greater agricultural sustainability by reducing the need for chemical inputs and enhancing soil health.

Multiplex genome editing and stacking multiple alleles with CRISPR/Cas can accelerate plant genetic improvement. However, the emergence of CRISPR crops has sparked international debates on regulation, risks, and differences between CRISPR-edited crops and GMOs. CRISPR has significantly reduced the cost of producing genome-edited crops, reshaping agriculture with precisely edited varieties (Bartkowski et al., 2018). Despite the economic benefits, concerns remain about unintended genome modifications and regulatory oversight due to the non-specific binding of sgRNA. The debate over legal, ethical, and policy issues surrounding CRISPR crops continues, with differing regulatory frameworks in different countries (Kondo and Taguchi, 2022). The international community is grappling with questions about regulatory oversight, safety data requirements, and the distinction between CRISPR-edited crops and GMOs (Zannoni, 2019). The regulatory landscape for CRISPR-edited crops is complex, with varying approaches to assessing and regulating these products. Developers and investors must navigate regulatory requirements before investing in CRISPR-edited crops. The choice of reagents and delivery methods for CRISPR/Cas systems can impact the final products and regulatory requirements. Understanding these factors is crucial for ensuring the acceptance and regulation of CRISPR-edited crops in the market (Ahmad M. et al., 2021).

### Regulatory aspects and biosafety concerns

Conventional mutagenesis and genetic engineering involve random genetic modifications that can lead to unintended changes in the genome, potentially disrupting genes or regulatory elements. This often requires extensive screening and regulatory approval (Monarkh, 2020). New genome editing (GE) technologies possessing site-directed nucleases (SDNs) offer precise control over random genome modifications and mutations. Genome editing techniques, such as ZFNs, TALENs, and CRISPR/Cas, allow for targeted changes in the genome, resulting in cost-effective and efficient trait introduction without the need for exogenous genetic material.

**FIGURE 4:**
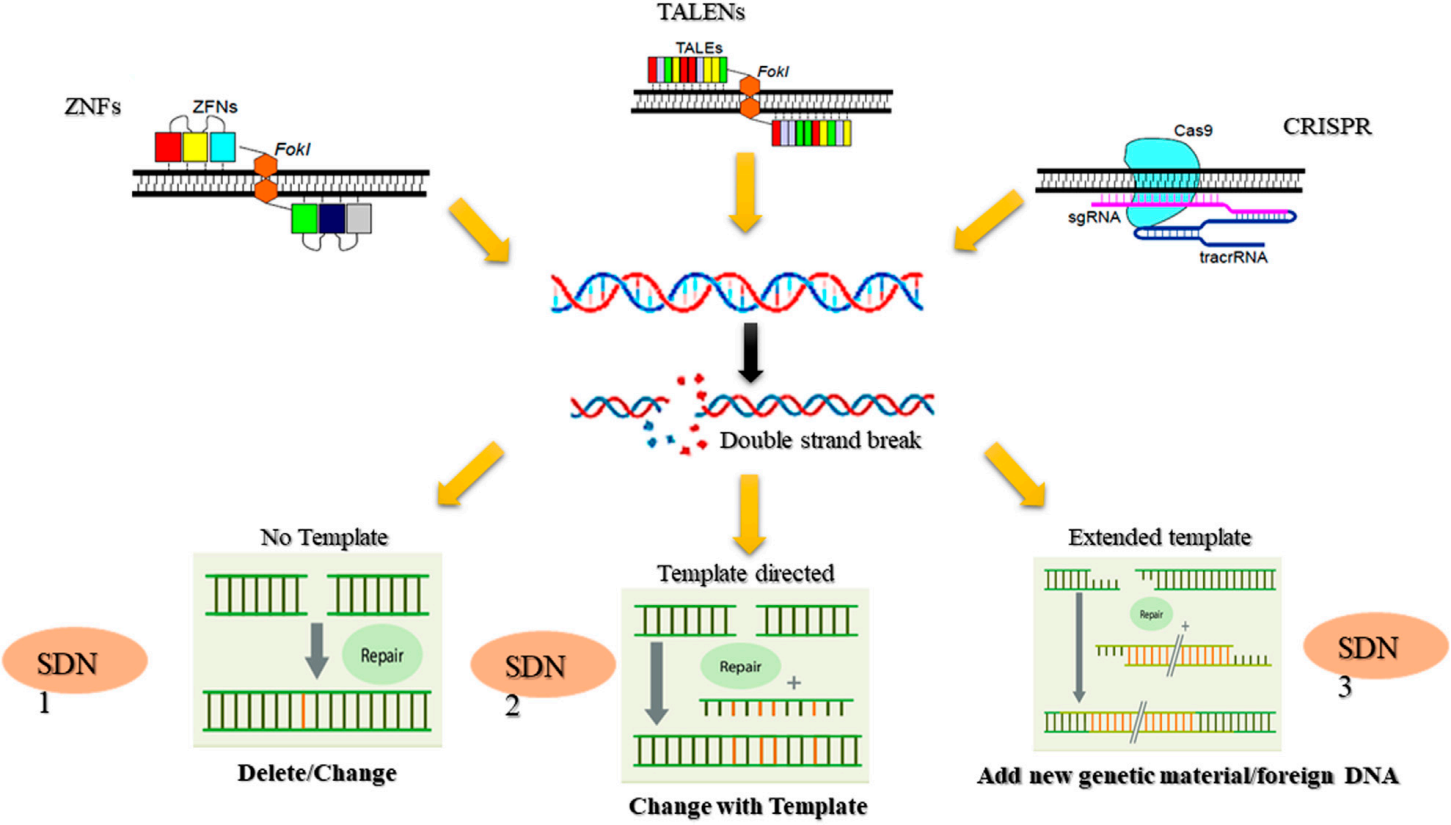
Genome editing and mechanisms using different tools, showing the occurrence of SDN1, SDN2, and SDN3 events.

Genome-edited mutants are classified into three types—SDN1, SDN2, and SDN3 (Figure 4), based on the nature of genome modifications and double-strand break (DSB) repair outcomes (Zannoni, 2019) SDN1 mutants involve precise point mutations that disrupt or silence genes without requiring a repair template. Although off-target effects in SDN1 mutants are debated, the resulting changes are similar to natural mutations (Zannoni, 2019; Sprink et al., 2016). SDN2 mutants, on the other hand, utilize a repair template to modify one or a few bases at a DSB site, yielding outcomes that mimic natural mutations and can be achieved with traditional breeding. However, SDN2 is limited for larger insertions due to its reliance on microhomology-mediated end joining (Verma et al., 2020). SDN3 mutants use a repair template with homologous regions of at least 500 base pairs flanking the DSB, enabling the insertion of new sequences, similar to transgenic or cisgenic approaches. Nonetheless, SDN3 applications in plants face challenges due to the low efficiency of homologous recombination (HDR), making them complex and less efficient (Verma et al., 2020; Podevin et al., 2013; Van de Wiel et al., 2017).

The commercial use of genome editing technologies is prejudiced by national legislation in genetic engineering and biotechnology, as well as facing the obstacle of consumer acceptance. Genome editing techniques in plant breeding have evolved, with CRISPR/Cas systems being widely adopted. The rapid development of genome editing poses challenges to existing regulations regimes worldwide. Current regulations may differentiate between genome editing techniques (SDN-1, SDN-2, SDN-3) and the use of foreign DNA or sequence templates in the editing process (Kulcsár et al., 2020).

Public concerns about environmental and health risks drive the need for consistent global regulations. Public acceptance of genetically modified crops can be improved through education, transparent regulatory processes, and ongoing risk assessments (Eckerstorfer et al., 2019). The CRISPR/Cas9 system offers a promising approach to developing improved plant varieties with minimal concerns. Transgene-free crops produced by CRISPR/Cas9 may bypass GMO regulations and do not require isolated field tests or labeling in some countries. Regulatory approaches for genetically engineered crops should balance safety, legal definitions, and public acceptance (Hessels et al., 2009). Decision-makers should consider the economic impact and social perceptions of handling genome-edited products under different regulatory scenarios to promote innovation in agriculture while ensuring sustainability. Regulatory policies (Table 2) need to prioritize improving food security and dietary health without compromising environmental or ethical values (Friedman et al., 2023).

**TABLE 2:** Regulatory aspects of genome-edited crops.

Sr No.	Country	Regulatory agencies	Approved genome edited crops	Regulation governing the release of gene-edited crops	References
1	US	USDA, APHIS, FDA, and EPA	Corn Tomato Soybean Mushroom Flax Non-browning apple	Coordinated Framework for Regulation of Biotechnology, New SECURE rules (2020)	Lombardo and Grando (2020)
2	Canada	Canadian Food Inspection Agency (CFIA)	Non-browning Potato Herbicide-resistant canola	Directive 94–08 (Dir 94–08) Assessment Criteria for Determining Environmental Safety of Plants with Novel Traits	Lombardo and Grando (2020)
3	Argentina	Argentine Biosafety Commission (CONABIA)	HB4 drought-resistant wheat	Resolution No. 173/15 (2015)	Lombardo and Grando (2020)
4	Brazil	National Technical Commission for Biosafety (CTNBio)	No approved crops	Normative Resolution No. 16 (2018)	Lombardo and Grando (2020)
5	Chile	Ministry of Agricultural and Livestock Services (SAG)	No approved crops	Introduction of methodological procedure (2017)	Haque et al. (2018)
6	Columbia	Colombian Agricutural Institute (ICA)	No approved crops	Resolution No. 00029299 (2019)	Haque et al. (2018)
7	Honduras	National Committee of Biotechnology and Biosecurity (NCBB)	No approved crops	Agreement SENASA 008–2019 (2019)	Haque et al. (2018)
8	Australia	Food Standards Australia New Zealand (FSANZ)	No approved crops	Gene Technology Act (Measures No. 1) to regulations (2019)	Haque et al. (2018)
9	China	National Biosafety Committee (NBC) Ministry of Agriculture and Rural Affairs (MARA)	No approved crops	Administrative Rules for the safety of agricultural GMOs	Haque et al. (2018)
10	India	Indian Ministry of Science and Technology (2020) Engineering Appraisal Committee (GEAC)	No approved crops	Regulatory Framework and Guidelines for Risk Assessment (2020)	Lombardo and Grando (2020)
11	Japan	The Ministry of Agriculture, Forestry and Fisheries (MAFF)	Tomato	GMOs, as defined under the Cartagena Act (2019)	Dima and Inzé (2021)
12	New Zealand	Food Standards Australia New Zealand (FSANZ)	No approved crops	Hazardous Substances and New Organisms Act (1998) after court decision NZHC 1067 (2014)	Bratlie et al. (2019)
13	Pakistan	Food Standards Australia New Zealand (FSANZ)	No approved crops	Pakistan Biosafety Rules, 2005	Shukla et al. (2018)
14	Spain and Portugal		No approved crops	Directive 18/2001/EC (2001) after a court decision in case C-528/16	Dima and Inzé (2021)

Increasing public acceptance of transgene-free crops can be achieved by raising awareness about CRISPR-based crops, building trust in safety regulations and developers, and clearly comparing risks and benefits (Ishii and Araki, 2016). The CRISPR/Cas9 system is considered the most effective method for developing improved plant varieties with minimal concerns. To address off-target issues, using the CRISPR system in the form of a rRNP and utilizing high-fidelity CRISPR variants can help minimize off-target effects (Kleinstiver et al., 2016). Modern technologies like whole genome sequencing can be used to assess potential off-target effects before creating genome-edited organisms. Transgene-free plants produced through CRISPR/Cas9 do not contain foreign DNA in the final product, potentially allowing them to bypass GMO regulations (Zetsche et al., 2017; Kim et al., 2017). This eliminates concerns about transgene flow to non-target species isolated field tests and GMO labeling requirements. However, in some countries, labelling of GM ingredients is mandatory, which can both build public trust and create resistance among certain consumer groups (Ishii and Araki, 2016; Voytas and Gao, 2014). For instance, a field trial of GM grapevine grafting in France faced disruptions from activists despite legal approval. The European Plant Sciences Organization has called for separating safety assessments and environmental risks from the labeling of genome-edited organisms to address these challenges (Zetsche et al., 2017).

DNA tagging in the genome of crops for the cultivation and marketing of genetically engineered organisms (GEOs) has been proposed (Ishii and Araki, 2016). However, this process involves additional gene modification steps and new GMO regulations, increasing costs for developers and companies (Duensing et al., 2018). Establishing appropriate regulatory guidelines is crucial to ensure the safety and legal definitions of genetically engineered crops. Clear regulatory rules can enhance public acceptance of GEOs, promote innovation in agriculture, and facilitate international trade (Whelan and Lema, 2015). Decision-makers should consider the economic impact of different regulatory scenarios for genome-edited products to anticipate social perceptions (Duensing et al., 2018). Political decisions should align with scientific recommendations to prevent excessive regulation that could hinder agricultural innovation and sustainability (Whelan and Lema, 2015). Regulatory policies for GEOs should emphasize the goal of enhancing food security and promoting a healthier diet while respecting environmental, religious, and ethical considerations.

### Potential impact on food security and sustainable agriculture

Agriculture today faces newer challenges exacerbated by genetic erosion, the narrow genetic base of commercial crops and environmental degradation. There is an urgentneed to make agriculture more resilient and sustainable while still continuing to develop. The Agriculture sector is facing newer challenges like narrow gene pool and genetic erosion among commercial crops and environmental challenges, etc. This would generate the need to make agriculture more resilient and sustainable by developing high-yielding, stress-tolerant, and climate-smart varieties. The CRISPR gene editing technologies are being harnessed and improved avariety of cereals, legumes, fruits, andunderutilized dryland crops. The CRISPR technologyhas the potential to enhance crop productivity, climate resilience, and nutritional value, and address the global challenges related to food security and sustainable agriculture.

The prospects of Biodiversity are potentially utilizedto improve food and agriculture and mitigate the crisis of hunger and malnutrition. A diverse gene pool in crops can provide valuable traits for improving crop resilience, yield, and nutritional quality. Besides, it allows natural systems to better withstand and recover from environmental stresses, such as diseases, pests, and climate change. However, the decline of natural resources, including crop diversity, alarming the loss of gene pools and may lose the future climate-smart breeding prospects of crop varieties (Lenka et al., 2020). Considering the declining biodiversity resources, serious attention has been paid to food security and the nutrition status of available diets. To ensure the demand of healthy food, there is an urgent need to of biofortification of fruits, vegetables, and cereals with enriched nutritional compounds such as vitamins, amino acids, antioxidants, proteins, minerals and fatty acids, etc.

Current strategies for the improvement of crop varieties for changing climatic conditions include traditional and molecular breeding methods, speed breeding, nanotechnology, mineral fertilization, and transgenic technologies. Recent modern gene editing technologies, such as CRISPR/Cas9, offer highly precise genetic modifications by targeting specific genes for alteration or replacement. This allows for the development of crops with improved traits without introducing foreign genes. CRISPR technology is utilized potentially in crops and is being utilized to improve crop varieties withrespect to nutritional quality, yield, disease and pest resistance, consumer acceptance, economic concerns, and environmental suitability, etc. Novel ARGOS8 edited variants of maize generated through CRISPR/Cas9 showed higher grain yield under drought stress conditions (Shi et al., 2017a; and b). The gene and base-editing strategies has precisely edited granule bound starch synthase gene (*StGBSSI)* gene in the tetraploid potato and with impaired amylose biosynthesis (Veillet et al., 2019).

### Future challenges and perspectives

With the assistance of AI and molecular engineering tools, CRISPR-based gene editing is gradually turning into a third-generation CRISPR-edited crop. The technology is showing tremendous potential in medical, agricultural, and environmental sciences. Particularly in agriculture, it has laid its footprint by generating broad-spectrum resistance against multiple pathogens, nutritional enhancement, etc. The false flax (C. sativa) exhibited enhanced omega-3 oil and was commercially released in the USA, showingrecord-breaking market space (Waltz, 2018). CRISPR modification has been extensively used in crop improvement by *de novo* meristem induction, targeting and editing efficiency can be enhanced by use of temperature-tolerant CRISPR/LbCas12a, largeerDNAinsertions with precision,use of heat-inducible CRISPR system in maize and breaking of genetic linkage via somatic chromosome engineering, etc., (Zaidi et al., 2020). This is an indicative of CRISPR based gene editing crop that has future potential.

Moreover, the other side of this technology has shown several concerns, particularly less specificity, genome complexity, editing efficiency, off-target effects, regulatory concerns, absence of testing methods, and lack of efficient delivery vectors and methods. In the absence of a transgene/foreign gene, the European Union (EU) is still treating the genome-edited crop as transgenic (Custers et al., 2019). The regulatory landscape for gene-edited crops varies globally. Some regions have adopted more permissive policies for gene-edited crops compared to genetically modified organisms (GMOs), reflecting evolving attitudes toward these technologies. Thusabsence of universal regulatory framework is also a concern affecting the research, cultivation, and marketing of gene-editedproducts. It warrants the need forcomprehensive plans for gene-edited crops from scientists and policymakers.

Apart from this, technological bottleneck, pertaining less to HDR efficiency affects gene replacement or deletions and large chromosomal segments. Third-generation AI-assisted gene editing methods would enable theprediction of on- and off-target edits, designing more powerful genome editors with increased editing efficiency, andaccelerating the pace of implementing safe agricultural use. Even third-generation CRISPR strategies are being introduced to engineer next-generation CRISPR crops, theystill facing the challenges of off-target edits, regulatory concerns, requirement of efficient delivery methods, recalcitrancy during transformation, requirement of extensive field trial for testing the performance.

## Conclusion

In conclusion, genome editing technologies, particularly CRISPR/Cas9, present powerful tools for the modulation of positive regulator genes to enhance climate resilience in crops. By targeting key regulatory genes and signaling pathways involved in stress tolerance mechanisms, researchers can develop crop varieties better equipped to withstand the multifaceted challenges posed by climate change. However, a holistic approach that integrates epigenetic regulation, small RNA-mediated processes, and plant-microbiome interactions is crucial for maximizing the potential of these technologies in ensuring global food security in the face of rapidly changing environmental conditions.
